# The multiple functions of the numerous *Chlamydia trachomatis* secreted proteins: the tip of the iceberg

**DOI:** 10.15698/mic2019.09.691

**Published:** 2019-08-21

**Authors:** Joana N. Bugalhão, Luís Jaime Mota

**Affiliations:** 1 UCIBIO, Departamento de Ciências da Vida, Faculdade de Ciências e Tecnologia, Universidade NOVA de Lisboa, Caparica, Portugal.

**Keywords:** host-pathogen interactions, Chlamydia trachomatis, protein secretion, type III secretion, effectors

## Abstract

*Chlamydia trachomatis* serovars are obligate intracellular bacterial pathogens mainly causing ocular and urogenital infections that affect millions of people worldwide and which can lead to blindness or sterility. They reside and multiply intracellularly within a membrane-bound vacuolar compartment, known as inclusion, and are characterized by a developmental cycle involving two morphologically and physiologically distinct chlamydial forms. Completion of the developmental cycle involves the secretion of > 70 *C. trachomatis* proteins that function in the host cell cytoplasm and nucleus, in the inclusion membrane and lumen, and in the extracellular milieu. These proteins can, for example, interfere with the host cell cytoskeleton, vesicular and non-vesicular transport, metabolism, and immune signalling. Generally, this promotes *C. trachomatis* invasion into, and escape from, host cells, the acquisition of nutrients by the chlamydiae, and evasion of cell-autonomous, humoral and cellular innate immunity. Here, we present an in-depth review on the current knowledge and outstanding questions about these *C. trachomatis* secreted proteins.

## INTRODUCTION

*Chlamydia trachomatis* serovars are human pathogens causing mostly ocular and genital infections [1, 2]. These infections affect millions of people worldwide and if left untreated can lead to blindness or sterility. *C. trachomatis* strains comprise three biovars, which can be further divided into 15 main serovars, based on antigenic variation of the major outer membrane protein (MOMP): the trachoma biovar (serovars A-C); the genital biovar (serovars D-K); and the lymphogranuloma venereum (LGV) biovar (serovars L1-L3). Most *C. trachomatis* infections are caused by genital strains, but studies on host cell-*C. trachomatis* interactions are usually performed with a prototype serovar L2 strain.

*C. trachomatis* is member of a Phylum (*Chlamydiae*) of Gram-negative bacteria, comprising one Class (*Chlamydiia*) and one Order (*Chlamydiales*), characterised by obligate growth within eukaryotic cells and including species that infect vertebrates, invertebrates, and eukaryotic microorganisms such as amoeba [3]. Among the *Chlamydiales*, the *Chlamydiacea* Family currently consists of 16 *Chlamydia* species [4], including *C. trachomatis*. In addition, the *Chlamydiacea* comprises *C. pneumoniae*, causing pulmonary infections in humans, and pathogens of a wide range of non-human vertebrates. Among the latter, *C. abortus, C. caviae, C. felis*, and *C. psittacci* have zoonotic potential.

**Figure 1:**
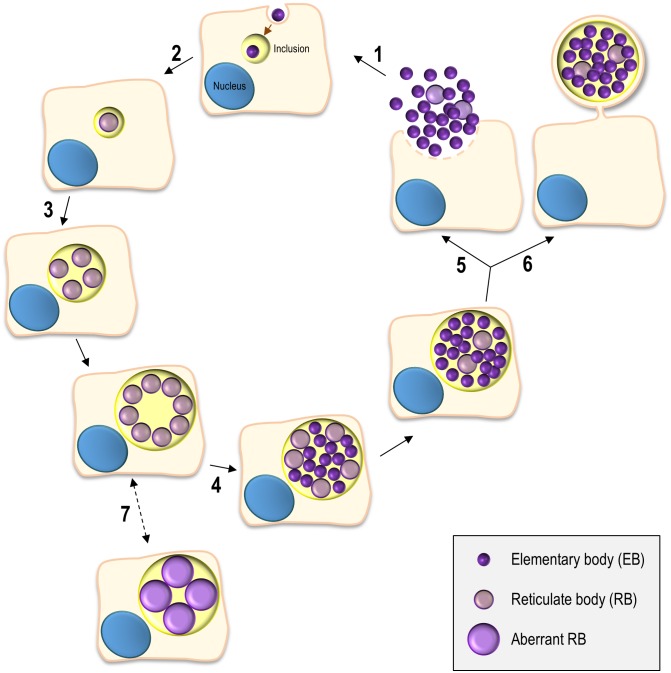
FIGURE 1: The chlamydial developmental cycle. (1) Adhesion to host cells by *C. trachomatis* EBs released from previously infected cells triggers the delivery of T3S effectors that overall mediate actin rearrangements resulting in chlamydial invasion, inhibition of interaction with the endocytic pathway, and modulation of host cell survival and immune signalling (~ 0-2 h post-infection). (2) The nascent inclusion segregates from the phagolysosomal pathway, the EBs differentiate into RBs, and the inclusion migrates along microtubules to a perinuclear centrosomal region (~ 2-6 h post-infection). (3) The RBs start replicating exponentially leading to a large inclusion occupying most of the host cell cytoplasm (~ 6-24 h post-infection). (4) The RBs re-differentiate asynchronously into EBs (~ 24-48 h post-infection). (5) & (6) The EBs (infectious progeny) and a few lasting RBs are released by host cell lysis (5) or extrusion (6) (~ 48-72 h post-infection). (7) Under certain stress conditions (antibiotics or cytokines) there is the reversible formation of aberrant RBs, a persister-like chlamydial form.

The *Chlamydiales* are characterized by a developmental cycle involving two distinct morphological forms, the small, infectious and non-replicative, elementary bodies (EBs; ~ 0.3 µm in diameter), and the larger, non-infectious and replicative, reticulate bodies (RBs; ~ 1 µm in diameter) (reviewed in [5, 6]). This cycle has been intensively studied in *C. trachomatis* using cultured cells as a model for the epithelial tissue encountered by this bacterium during *in vivo* infection and can take ~ 48-72 h, depending on the strain **(Figure 1)**. Adherence of EBs to the surface of host cells leads to chlamydial internalization and to the formation of a membrane-bound compartment, a *Chlamydia*-containing vacuole generally known as inclusion. About 2 h after internalization, the intravacuolar EBs start differentiating into RBs, which begin to replicate ~ 6 h post-infection. Multiple rounds of chlamydial replication result in a large inclusion occupying a significant part of the host cell cytoplasm. From ~24 to 48-72 h post-infection, RBs re-differentiate asynchronously into EBs. The inclusion is then filled with EBs (the infectious progeny), which after release from the host cell can infect neighbouring cells **(Figure 1)**.

*C. trachomatis* interferes with a wide range of host cell processes during its developmental cycle (reviewed in [6]; **Figure 1**). Subversion of host receptor-mediated signalling and of the actin cytoskeleton and its key regulators promotes chlamydial adherence and invasion of host cells. While intracellularly, *C. trachomatis* reshapes the protein and lipid composition of the inclusion membrane by selective interactions with molecules controlling and mediating host cell vesicular trafficking, segregating from the endolysosomal pathway and forming a unique and stable vacuolar compartment that localizes near the centrosome. The interaction with host cell vesicular and non-vesicular transport pathways also enables *C. trachomatis* to acquire nutrients and lipids required for its growth. In addition, among other aspects, intravacuolar *C. trachomatis* modulates host cell survival and death and the innate immune signalling. Finally, to exit from the host cell, *C. trachomatis* subverts the host cell cytoskeleton and calcium-signalling.

Completion of the developmental cycle and subversion of host cells processes by *C. trachomatis* involves the timely secretion of many chlamydial proteins. We will survey and discuss the knowledge on the identity and function of *C. trachomatis* secreted proteins that participate or might participate in the subversion of host cell processes. This knowledge has significantly increased in recent years because of developments in methods to genetically manipulate *C. trachomatis* [7–15] (and reviewed in [16]) that followed or paralleled the first description of a system to transform *C. trachomatis* [17].

## PROTEIN TRANSPORT SYSTEMS IN *C. TRACHOMATIS*

**Figure 2:**
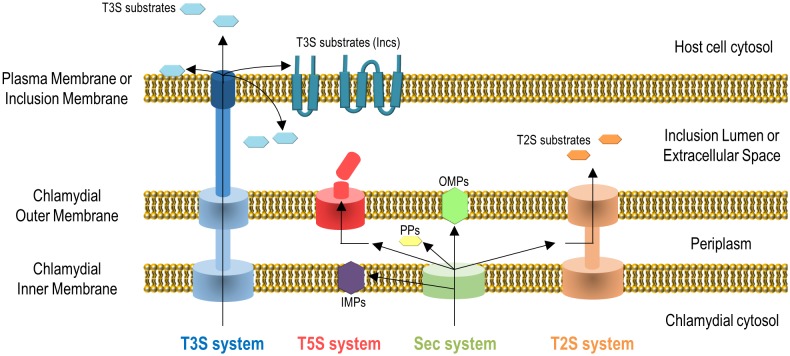
FIGURE 2: Secretion systems used by *C. trachomatis* to transport proteins to different aqueous and membranaceous chlamydial and host cell compartments. It is unclear how some chlamydial T3S substrates are transported into the inclusion lumen or how Incs insert into the inclusion membrane. IMPs, chlamydial inner membrane proteins; PPs, chlamydial periplasmic proteins; OMPs, chlamydial outer membrane proteins. See list of abbreviations and main text for details.

Gram-negative bacteria use several protein transport systems that are essential for interactions with other cells and with the extracellular environment [18–20]. In the case of *C. trachomatis*, its genome encodes the Sec system, a type II secretion (T2S) system, a type III secretion (T3S) system, Sec-exported polymorphic membrane proteins (Pmps) containing type V secretion (T5S) system/autotransporter signals, and several other outer membrane proteins [21, 22] **(Figure 2)**. The Sec- and T3S system-dependent transport of *C. trachomatis* proteins by heterologous bacteria [23, 24], the isolation and characterization of *C. trachomatis* mutants in the T2S system-associated ATPase [14] and in PmpD [25], and different biochemical, gene expression and proteomic analyses [26–29], showed the functionality and importance of all these protein transport systems for the *C. trachomatis* developmental cycle and for *Chlamydia*-host cell interactions. All together, these systems enable the delivery of chlamydial proteins to different aqueous and membranaceous compartments within bacterial and mammalian host cells **(Figure 2)**. The use in *C. trachomatis*-infected cells of small molecules reported to inhibit the Sec system and the T3S system also initially indicated the importance of these protein transport systems for chlamydiae [24, 30–32]. However, in the case of the use of small molecules that can inhibit T3S systems, they have been later shown to chelate iron [33] and to bind chlamydial protoporphyrinogen oxidase (HemG) [34]. Therefore, conclusions based on the use of these small molecules to define the importance of the T3S secretion pathway for *C. trachomatis* are questionable.

Chlamydial outer membrane proteins, such as the Pmps [35], outer membrane complex protein B (OmcB) [36], MOMP [37–39], or *C. trachomatis* adhesin 1 (Ctad1) [40] are important for the initial contact and adhesion of *C. trachomatis* with host cells [6], but they will not be further described here. We will focus on *C. trachomatis* proteins: (i) that localize in the inclusion membrane, functioning as effectors mediating the chlamydiae-host cell interaction; (ii) that localize and function as effectors in the host cell plasma membrane, cytoplasm or nucleus; (iii) that localize in the inclusion lumen, where they function and/or from where they might be transported into the inclusion membrane and/or host cell cytosol, or are released in the extracellular environment after chlamydial exit.

## IDENTIFICATION OF *C. TRACHOMATIS* INC PROTEINS AS T3S SUBSTRATES

The most prominent group of *Chlamydia* proteins mediating bacterial-host cell interaction are the inclusion membrane proteins (Incs) [6, 41]. Although the amino acid sequences of Incs from the same *Chlamydia* species are mostly unrelated to each other [42, 43], they are all characterized for localizing at the inclusion membrane and for at least one bilobed hydrophobic motif [6, 41]. Thirty-six bona fide Incs have been identified in *C. trachomatis* **(Table 1)**, and several more might exist [42, 43]. Other members of the *Chlamydiales*, including endosymbionts of free-living amoeba [44], also likely possess large numbers of Incs [42, 43].

**TABLE 1:** *C. trachomatis* known Inc proteins^a^.

**Inc protein (annotation/name)**			**Host cell protein targets^b^**	**Proposed functions**	**References**
**Strain D/UW3**	**Strain L2/434**	**General**			
CT005	CTL0260	IncV	VAPA/B	Formation of ER-inclusion MCS; non-vesicle lipid uptake by *C. trachomatis*.	[52, 59, 125, 189]
CT006	CTL0261	-	Unknown	Unknown.	[59]
CT101	CTL0356	MrcA	ITPR3	Promotion of chlamydial extrusion; localize at inclusion microdomains.	[52, 128, 130]
CT115	CTL0370	IncD	CERT	Formation of ER-inclusion MCS; non-vesicle lipid uptake by *C. trachomatis*.	[50, 51, 120]
CT116	CTL0371	IncE	SNX5/6	Modulation of retromer-dependent trafficking.	[50, 51, 59, 86–88]
CT117	CTL0372	IncF	Unknown	Heterophilic Inc-Inc interactions.	[50, 51, 59, 75]
CT118	CTL0373	IncG	14-3-3β	Unknown; associates with LDs.	[50, 51, 145, 221]
CT119	CTL0374	IncA	VAMP3/7/8	Homotypic inclusion fusion; regulation of host cell vesicular trafficking; associates with LDs.	[8, 48, 50, 51, 69, 74, 77, 78, 189, 220]
CT134	CTL0389	-	Unknown	Unknown.	[59]
CT135	CTL0390	-	Unknown	Important for chlamydial virulence in a mouse infection model.	[59, 149, 150]
CT147	CTL0402	-	Unknown	Unknown.	[50, 59, 65]
CT179	CTL0431	-	Unknown	Unknown.	[59]
CT192	CTL0444	-	Unknown	Unknown.	[59]
CT222	CTL0475	-	Unknown	Heterophilic Inc-Inc interactions; localizes at inclusion microdomains.	[52, 59, 75, 128]
CT223	CTL0476	IPAM	CEP170	Modulation of the microtubule network; inhibition of host cell cytokinesis; localizes at inclusion microdomains.	[49, 50, 52, 59, 109, 133]
CT224	CTL0477	-	Unknown	Inhibition of host cell cytokinesis; localizes at inclusion microdomains.	[52, 59, 133]
CT225	CTL0477A	-	Unknown	Inhibition of host cell cytokinesis.	[50, 52, 133]
CT226	CTL0478	-	Unknown	Unknown.	[50, 52, 56, 59]
CT227	CTL0479	-	Unknown	Unknown.	[52, 59]
CT228	CTL0480	-	MYP1	Inhibition of chlamydial extrusion; localizes at inclusion microdomains.	[50, 52, 138, 140]
CT229	CTL0481	CpoS	RABs	Control of inclusion membrane stability and/or host cell death, and of host cell vesicular trafficking.	[49, 50, 52, 59, 96–98, 100]
CT232	CTL0484	IncB	Unknown	Localizes at inclusion microdomains.	[50, 59, 128]
CT233	CTL0485	IncC	Unknown	Control of inclusion membrane stability; localizes at inclusion microdomains.	[49, 50, 59, 100]
CT249	CTL500A	-	Unknown	Unknown.	[50, 52, 57]
CT288	CTL0540	-	CCDC146	Localizes at inclusion microdomains.	[49, 50, 59, 135]
CT345	CTL0599	-	Unknown	Unknown.	[59]
CT358	CTL0612	-	Unknown	Unknown.	[50]
CT383	CTL0639	-	Unknown	Modulation of inclusion membrane stability.	[59, 100]
CT440	CTL0699	-	Unknown	Unknown.	[50]
CT442	CTL0701	CrpA	Unknown	Unknown.	[49, 50, 53, 59]
CT449	CTL0709	-	Unknown	Unknown.	[59]
CT483	CTL0744	-	Unknown	Unknown.	[52]
CT565	CTL0828	-	Unknown	Unknown.	[52]
CT618	CTL0882	-	Unknown	Associates with LDs.	[50, 54, 221]
CT813	CTL0184	InaC	14-3-3 proteins, ARF1/4, VAMP7/8	Modulation of post-translational modification of microtubules, and of F-actin and Golgi redistribution around the inclusion.	[15, 50, 52, 55, 74, 112]
CT850	CTL0223	-	DYNLT1	Inclusion positioning at the centrosomal region; localizes at inclusion microdomains.	[52, 128]

a Proteins with the characteristic hydrophobic bilobal domain and which have been experimentally detected at the inclusion membrane. See list of abbreviations and main text for abbreviations and protein nomenclature, respectively.

b Only interactions of *C. trachomatis* Incs (and not of Incs from other *Chlamydia* species) with host cell proteins were considered; while potential interactions between several *C. trachomatis* Incs and many human proteins have been described by large scale proteomics [85], only those further validated are specified in Table 1.

The first report of the identification of an Inc dates to 1995 [45]. By using sera of guinea pigs infected with *C. psittaci* or immunized with killed EBs, proteins absent in purified EBs or in uninfected cells were localized at the inclusion membrane by immunofluorescence (IF) microscopy [46]. The same sera were used to screen an expression library of *C. psittaci* DNA, and this led to the identification of a gene encoding an inclusion membrane protein, named IncA [45]. A similar approach led to the identification of *C. psittaci* IncB and IncC [47]. Data from the first genome of *C. trachomatis*, released in 1998 [21], revealed orthologues of Incs A, B, and C [47, 48], which were shown to localize at the inclusion membrane of *C. trachomatis* [48–50]. Moreover, sera obtained from rabbits immunized with the membrane fraction of HeLa cells infected by *C. trachomatis* led to the identification of Incs D, E, F, and G [51].

After the identification of Incs A, B and C, the characteristic bilobed hydrophobic region was used to search the genome of *C. trachomatis* for genes that could encode additional Incs [49]. By raising antibodies against the putative *C. trachomatis* Incs and showing by IF microscopy that they localize at the inclusion membrane, the bilobed hydrophobic region was defined as a characteristic motif of Incs and a determinant of their localization [49]. In subsequent studies, similar or related approaches were used to identify additional *C. trachomatis* Incs [50, 52–57], and to predict more putative Incs by bioinformatics [42, 43]. The development of tools to genetically manipulate *C. trachomatis* [7, 9, 17, 58], helped in the identification of additional bona fide Incs [59], but it also revealed several putative Incs that did not localize at the inclusion membrane [59]. Different timings and levels of expression of the tetracycline-inducible system used could affect protein localization; but it is also possible that some of the bioinformatically predicted Incs do not localize at the inclusion membrane.

The lack of a cleavable Sec signal peptide on the first identified Incs and the discovery of homologues of T3S system genes in *Chlamydia* [21, 60], suggested that Incs could be T3S substrates [47, 49, 51, 61]. Methods for genetic manipulation of *Chlamydia* were unavailable at the time, but it had been shown that chlamydial proteins could be type III secreted by heterologous bacteria [23]. Using this methodology, the N-terminal region of Incs was shown to contain a signal capable of mediating secretion of hybrid proteins by the T3S system of *Shigella flexneri* [62, 63], which established the concept of Incs as T3S substrates. In subsequent studies several *C. trachomatis* Inc proteins were confirmed as T3S substrates [42, 59, 64].

## THE FUNCTIONS OF *C. TRACHOMATIS* INC PROTEINS

Analyses of *C. trachomatis* gene expression revealed at least three classes of Incs depending on their corresponding messenger RNA (mRNA) levels during the chlamydial developmental cycle: early-cycle Incs (highest mRNA levels between ~2-6 h post-infection); mid-cycle Incs (highest mRNA levels between 6-20 h post-infection); late-cycle Incs (highest mRNA levels after ~20 h post-infection) [52, 64–66]. This suggested roles of Incs at distinct stages of the chlamydial developmental cycle, an idea which is generally being confirmed as the host cell subverting functions of Incs are being characterized.

### Incs modulating host cell vesicular trafficking

**Figure 3:**
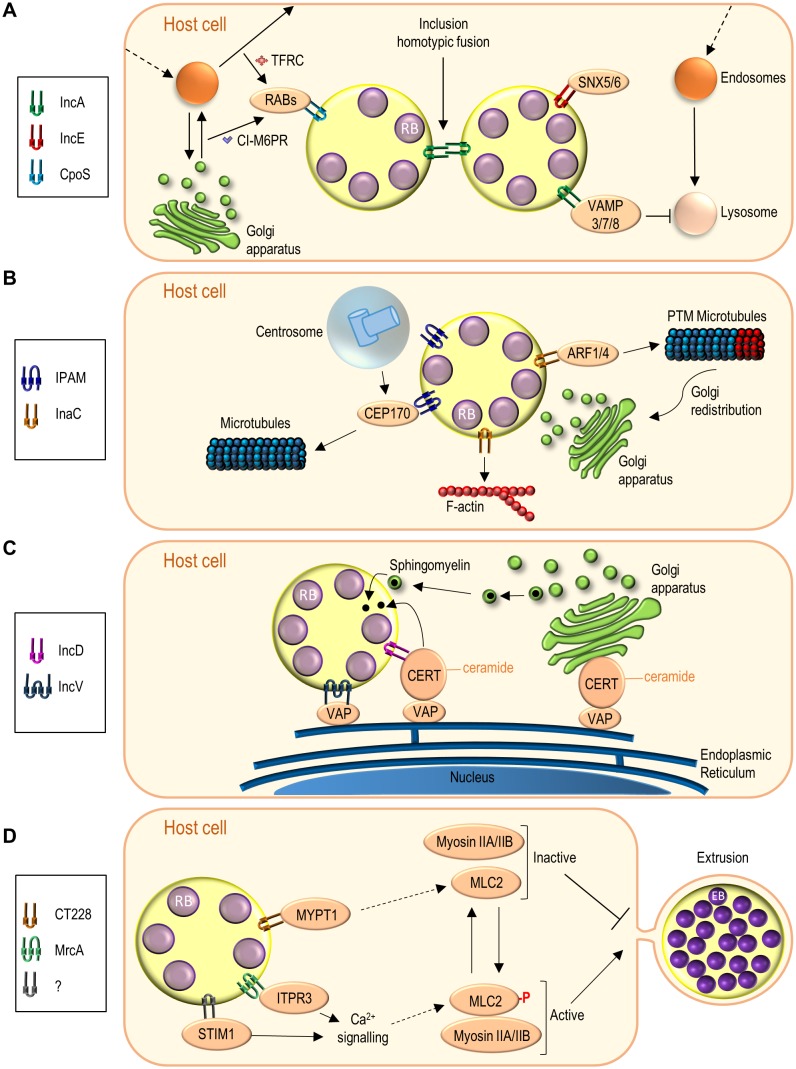
FIGURE 3: Functions of *C. trachomatis* Incs effectors. **(A)** Incs subverting host cell vesicular transport (IncA, IncE, CpoS). **(B)** Incs interfering with microfilaments and microtubules (IPAM, InaC), and mediating Golgi redistribution around the inclusion (InaC); PTM, post-translationally modified. **(C)** Incs participating in ER-inclusion MCSs (IncD, IncV). **(D)** Incs modulating chlamydial extrusion by subverting Ca^2+^ signalling and myosin II function (CT228, MrcA). An unknown Inc possibly recruits STIM1 to the inclusion membrane. The number of transmembrane domains illustrated for each Inc is based on [42]. See list of abbreviations, main text, and **Table 1** for details.

As an intravacuolar pathogen, *C. trachomatis* manipulates host cell vesicular trafficking at least to avoid the phagolysosomal route leading to bacterial destruction and to intercept vesicles containing nutrients required for bacterial growth and inclusion expansion [67]. Given their localization, Incs are natural candidates for subversion of vesicular trafficking, and, until now, three *C. trachomatis* Incs (IncA, IncE and CpoS (*Chlamydia* promoter of survival); **Table 1**) have been shown to be directly involved **(Figure 3A)**.

#### Homotypic fusion between inclusions

*C. trachomatis* normally resides and multiplies within a single inclusion containing several chlamydiae. In contrast, *C. trachomatis*-infected cells microinjected with anti-IncA antibodies show multiple inclusions within a single host cell [68]. Furthermore, *C. trachomatis* clinical isolates residing in multiple inclusions within infected cells have mutations in *incA* and lack the IncA protein in the inclusion membrane [69, 70]. This led to the concept that IncA mediates the fusion between inclusions within infected cells **(Figure 3A)**, which was confirmed when *C. trachomatis incA* insertional mutants were generated and characterized [8, 71].

In eukaryotic cells, membrane fusion requires interactions between soluble N-ethylmaleimide-sensitive factor attachment protein receptors (SNAREs) in opposing lipid bilayers [72]. Bioinformatics disclosed the presence of two SNARE-like domains (SLD1 and SLD2) in IncA [73, 74]. Yeast and bacterial two-hybrid, and different biochemical experiments, revealed homotypic IncA:IncA interactions [68, 73, 75–77]. The C-terminally-located SLD2 of IncA is required for the homotypic interactions [75, 77], and SLD1 and part of SLD2 are needed for fusion between inclusions [71, 76, 77]. This indicates that SNARE-like IncA:IncA interactions mediate the fusion between inclusions **(Figure 3A)**. However, the recent determination by X-ray crystallography of the three-dimensional (3D) structure of almost all the cytosolic region of IncA [78], indicates that its structure differs from SNAREs and resembles instead a domain of the Huntingtin-interacting protein-1 related (HIP1R) that mediates associations between actin and clathrin-coated structures [78, 79].

*C. trachomatis* IncA can also interact with mammalian SNAREs (vesicle-associated membrane proteins (VAMPs) 3, 7, and 8), which are recruited to the periphery of the inclu-sion [74]. The interaction depends on the SLDs of IncA and the recruitment of the SNAREs is reduced in cells infected by *C. trachomatis* lacking IncA [74]. Moreover, IncA inhibits endocytic SNARE-mediated fusion and both SLD1 and SLD2 can perform this activity [77, 80]. This suggests that IncA has an inhibitory action on the SNAREs it interacts with **(Figure 3A)**.

In summary, IncA mediates inclusion fusion and has the capacity to inhibit endocytic membrane fusion **(Figure 3A)**. Because *incA* is expressed at mid-cycle [52, 64], this ability to inhibit fusion with endocytic vesicles should not be relevant for the initial segregation of the nascent inclusion from the endolysosomal pathway, but could however help to protect the inclusion from unwanted vesicle fusion. Epidemiological and clinical studies indicated that infection with *C. trachomatis* isolates with *incA* mutations results in milder symptoms and reduced bacterial load [81]. Moreover, a non-fusogenic clinical strain with an *incA* mutation revealed slower growth in cultured cells [82]. However, a more recently characterized *C. trachomatis incA* null-mutant strain reveals no growth defect in cultured cells [71]. This suggests a possible functional redundancy of IncA with other unidentified *C. trachomatis* proteins that should also contribute to inhibition of fusion with endocytic vesicles.

#### Subversion of the retromer

The retromer is a protein complex, including at least one sorting nexin (SNX), which recycles cargo from endosomes to the plasma membrane or to the *trans*-Golgi network (TGN) [83]. Proteomics of isolated *C. trachomatis* inclusions and a screen for human proteins interacting with *C. trachomatis* Incs both disclosed the importance of the retromer in chlamydial host cell infections [84, 85]. Several SNXs are enriched in the inclusion [84], and IncE binds SNXs 5 and 6 [85] **(Figure 3A)**. The IncE:SNX5 interaction has been studied at a structural level [86–88]. This eventually revealed that IncE competes with cation-independent mannose 6-phosphate receptor (CI-M6PR) for binding to SNX5, and that the SNX5:CI-M6PR interaction is inhibited during host cell infection by *C. trachomatis* [86–88]. M6PRs are involved in transport of newly synthesized lysosomal enzymes from the TGN to endosomes, and their subsequent recycling back to the Golgi requires the retromer [89]. As depletion of retromer components, and in particular of SNX5, increases the production of chlamydial infectious progeny [84, 85], this indicates that retromer restricts *C. trachomatis* infection and that IncE might subvert retromer and lysosomal function by binding SNXs 5 and 6. This IncE-dependent subversion of the retromer might, for example, enable *C. trachomatis* to acquire nutrients, or to avoid lysosome- or autophagy-mediated killing [90].

#### Targeting of RAB GTPases

RAB GTPases are master regulators of eukaryotic vesicular trafficking [91], and several of them (RABs 1, 4, 6, 8, 10, 11, 14, 34, 35, 39a, 39b) have been reported to localize at, or in close proximity of, the *C. trachomatis* inclusion membrane [92–96]. Despite this, until now, only one *C. trachomatis* protein, Inc CpoS **(Table 1)**, has been shown to bind and modulate the function of RABs **(Figure 3A)**.

Yeast two-hybrid and pull-down experiments first revealed an interaction between CpoS and RAB4 [97]. More recently, co-immunoprecipitation (co-IP) experiments from mammalian cells ectopically expressing CpoS, or infected by *C. trachomatis* encoding epitope-tagged CpoS, revealed or confirmed interactions of this Inc with RABs 1, 2, 4, 6, 8, 10, 14, 18, 33, 34, and 35 [85, 96, 98]. Furthermore, recruitment to the periphery of the inclusion of all these RABs was impaired in cells infected by *C. trachomatis cpoS* null mutants [96, 98], and depletion of RABs 4, 6, 14, or 35 reduced the production of infectious progeny [93, 96, 99].

Expression of CpoS is toxic to yeast cells, which led to the identification of proteins related with clathrin-coated vesicles that when overexpressed suppressed CpoS-mediated toxicity [96]. Somewhat in line with this, CpoS is required for the accumulation of transferrin (the transferrin receptor (TFRC) traffics through clathrin-dependent transport from the plasma membrane to endosomes and by subsequent RAB-dependent recycling) and of CI-M6PR (this receptor traffics through clathrin- and RAB-dependent transport from the TGN to endosomes and subsequent RAB-dependent recycling) near the inclusion membrane [96] **(Figure 3A)**. Furthermore, this accumulation of transferrin is dependent on RABs 4 and 35 [96]. In summary, by targeting multiple RABs, CpoS is an important regulator of host cell vesicular trafficking in *C. trachomatis*-infected cells. Accordingly, *cpoS C. trachomatis* mutants are attenuated in the generation of infectious progeny in cultured cells and in mice infection models [98, 100]. The activity of CpoS might be important for acquisition of nutrients, avoidance of fusion of the inclusion with lysosomes, stability of the inclusion membrane, and/or modulation of host cell death (as further described below).

### Incs controlling inclusion membrane stability and host cell death

Intracellular pathogens must ensure the integrity of their replicative niche and therefore they often inhibit host cell death [101]. Intravacuolar pathogens also need to control the stability of the membrane of the pathogen-containing vacuole [102], because cytosolic release of the pathogen can lead to host cell death [103]. *C. trachomatis* Incs CpoS, IncC and CT383 **(Table 1)** have been reported to control these processes in *Chlamydia*-infected cells.

CpoS derives its name from the observation that cells infected by *C. trachomatis cpoS* null mutants die much more frequently than cells infected by the wild-type strain [98]. This has been described in two separate studies that diverge in the explanation for the cytotoxic effect of CpoS-deficient *C. trachomatis* [98, 100].

In one study, Weber *et al.* performed insertional mutagenesis of eleven *inc* genes eventually revealing that infection by *C. trachomatis cpoS, incC*, or *ct383* null mutants resulted in increased host cell death [100]. As the *cpoS* mutant [98, 100], the *incC* and *ct383* mutants were also defective for generation of infectious progeny in cultured cells and attenuated in a mouse infection model [100]. Because IF microscopy of cells infected by each of the three *inc* mutants revealed both multiple inclusions per infected cell and premature lysis of the inclusion membrane, the increased cytotoxicity was proposed to be a consequence of the release of the chlamydiae in the host cell cytosol [100]. Additional experiments suggested that the cytosolic release of each of the three *inc* mutant chlamydiae leads to autophagy-dependent host cell death [100].

In the study by Sixt *et al.* [98], infection by a *cpoS* mutant was shown to activate the stimulator of interferon genes (STING), leading to its re-localization from the endoplasmic reticulum (ER) to perinuclear vesicles and to the triggering of a signalling pathway that results in an enhanced interferon (IFN) response [98]. While host cell death promoted by infection with *cpoS* mutant *C. trachomatis* was reduced in STING-deficient cells, this reduction was not observed upon pharmacological inhibition of the transport of STING from the ER into perinuclear vesicles or of the downstream signalling pathway [98]. This indicates that the IFN response and host cell death promoted by CpoS-deficient *C. trachomatis* are independent processes [98]. Experiments with inhibitors of an ER calcium pump known to interact with STING suggested that the cytotoxicity associated with infection by CpoS-deficient *C. trachomatis* could be related with control of calcium pools in the ER [98].

In summary, in one model, lack of CpoS leads to inclusion lysis and autophagy-dependent host cell death [100], while in the other absence of CpoS does not significantly affect stability of the inclusion membrane but leads to the activation of host cell death that is partially dependent on STING [98].

### Incs modulating the Golgi and the host cell cytoskeleton

*C. trachomatis* manipulates and remodels the eukaryotic cytoskeleton (intermediate filaments, microfilaments, microtubules, and septins) at different stages of the developmental cycle [104–110], and promotes the redistribution of the Golgi complex around the inclusion [111]. Thus far, *C. trachomatis* Incs IPAM (inclusion protein acting on microtubules) and InaC (inclusion membrane protein for actin assembly) **(Table 1)** have been shown to be involved in the subversion of microtubules and microfilaments and in Golgi redistribution.

#### Microtubule remodelling

Host cell microtubules accumulate in a nest-like structure around the *C. trachomatis* inclusion, which suggested the involvement of an Inc [109]. IPAM was singled out as a candidate, based on the similarity of its primary structure with human centrosomal and microtubule-related proteins [109]. In infected cells, IPAM localizes at the inclusion membrane in patches near the centrosome [59, 109]. In uninfected cells, ectopically expressed IPAM associates with the centrosome and alters the organization of microtubules [109]. IPAM binds centrosomal protein 170 (CEP170) and this host cell protein is required for accumulation of microtubules around the inclusion, proper inclusion morphology, generation of infectious progeny, and for the effect of ectopically expressed IPAM on the organization of microtubules [109]. Thus, IPAM likely remodels microtubules in infected cells through CEP170 **(Figure 3B)**.

#### Actin remodelling and Golgi redistribution

Filamentous (F)-actin also accumulates around the inclusion [106]. A collection of chemically mutagenized *C. trachomatis* strains was used to screen for the chlamydial genes involved in this F-actin accumulation [15]. This led to the identification of the gene encoding InaC [15], which was then also shown to be necessary for Golgi redistribution around the inclusion [15] **(Figure 3B)**. Although experiments with drugs interfering with actin polymerization suggested that InaC-dependent remodelling of F-actin around the inclusion could be required for Golgi redistribution [15], analyses of cells infected by a *C. trachomatis* strain overexpressing InaC indicated that these are two independent processes [112].

Eukaryotic ADP ribosylating factors (ARFs) are small GTPases regulating vesicular trafficking, actin remodelling and the structure of the Golgi complex [113], and they are also targeted by *C. trachomatis*. InaC binds and recruits ARFs 1 and 4 to the periphery of the inclusion [15, 112]. Moreover, InaC mediates the activation of ARFs 1 and 4, and this leads to the induction of post-translational modifications of microtubules that promote Golgi redistribution around the inclusion [108, 112] **(Figure 3B)**. On the other hand, there is currently no evidence for a role of ARFs in F-actin remodelling mediated by InaC [112].

F-actin remodelling and Golgi redistribution have been suggested to stabilize the inclusion and to promote the acquisition of lipids [106, 111], respectively. However, *inaC* mutants do not display a defect in trafficking of sphingolipids to the inclusion [15, 112]. Furthermore, there are disparate observations regarding the ability of *C. trachomatis inaC* null mutants to generate infectious progeny in cultured cells, as a defect was observed with one mutant [112], but not with other two [15, 100]. Finally, as IncA, InaC possesses a SLD and can bind VAMPs 7 and 8 [74], but the significance of these interactions is unknown.

### Incs in ER-inclusion membrane contact sites

Eukaryotic organelles can interact through membrane contact sites (MCSs), corresponding to areas of close apposition between membranes involving tethering and functional protein complexes, but where membrane fusion does not occur [114]. Besides intercepting vesicular trafficking to obtain host cell lipids [115–118], *Chlamydia* can also obtain lipids through an ER-inclusion MCS. At least two *C. trachomatis* Incs (IncD and IncV; **Table 1**) are involved.

In mammalian cells, the transport of ceramide from the ER to the Golgi involves an MCS and is mediated by the ceramide transporter (CERT); ceramide is then converted into sphingomyelin by synthases in the Golgi [119] **(Figure 3C)**. A small-interfering RNA (siRNA) screen [120], and the observation that, although sphingomyelin is essential for chlamydial growth, blocking vesicular transport of sphingomyelin does not inhibit chlamydial replication [116, 121, 122], both eventually led to the identification of CERT as an important player in *C. trachomatis* growth [120, 122].

CERT, its ER binding partners (VAMP-associated protein A (VAPA) and VAPB), and host cell sphingomyelin synthases were shown to localize near the inclusion membrane by IF microscopy [120, 122]. Immunoelectron microscopy revealed the localization of CERT in the inclusion membrane presumably connecting with VAPB in nearby ER tubules, thus suggesting the concept of an ER-inclusion MCS [120]. Furthermore, depletion of CERT, VAPA/B, or sphingomyelin synthases reduced the generation of infectious progeny [120]. The physical proximity between the ER and the inclusion has also been revealed by electron tomography [123]. This included the identification of regions, termed pathogen synapses, where T3S system complexes connect the chlamydiae to the inclusion membrane specifically at the points of contact with the ER [123].

How is CERT recruited to the inclusion membrane? Immunoprecipitation from extracts of cells infected by *C. trachomatis* and ectopically expressing epitope-tagged CERT revealed an interaction with IncD that was further validated [120]. Furthermore, recruitment of CERT to the inclusion membrane is increased in cells infected by *C. trachomatis* overexpressing IncD [124]. Recruitment of VAPA/B to the periphery of the inclusion also correlates with IncD expression, but this occurs indirectly through CERT:VAPA/B interactions mediated by a two phenylalanines in an acidic tract (FFAT) motif in CERT [124]. In summary, ceramide is thought to be transported from the ER into the inclusion through a MCS involving a VAPA/B:CERT:IncD complex **(Figure 3C)**. In the inclusion, ceramide should be converted into sphingomyelin by a sphingomyelin synthase recruited to the inclusion membrane through a currently unknown mechanism [122].

Another *C. trachomatis* Inc (IncV) is a factor establishing ER-inclusion tethering [125]. An interaction between IncV and VAPA/B was first reported in a large-scale proteomics screen for human proteins interacting with *C. trachomatis* Incs [85]. The IncV:VAPA/B interaction was subsequently validated and shown to depend on FFAT motifs in IncV [125]. Recruitment of VAPA/B to the periphery of the inclusion is much increased in cells infected by *C. trachomatis* overexpressing IncV, and slightly reduced in cells infected by a *C. trachomatis incV* mutant [125]. Different experiments support that IncV mediates ER-inclusion tethering through its binding to VAPA/B [125] **(Figure 3C)**. A *C. trachomatis incV* mutant does not display a growth defect in cultured cells [100], seemingly suggesting that IncV is not essential for the presumed transport of ceramide into the inclusion by the VAPA/B:CERT:IncD complex in the ER-inclusion MCS. This would indicate that other chlamydial factors should also be involved in establishing ER-inclusion tethering. However, ceramide can also be delivered into the inclusion by vesicular transport and the negative impact of depleting CERT on infectious progeny might be explained by reasons other than the role of this host protein in the ER-inclusion MCS.

### Incs concentrated at inclusion microdomains and near the centrosome

About 2 h after invasion of host cells by *C. trachomatis*, the nascent inclusion migrates along microtubules towards the centrosome propelled by the minus end-directed microtubule dynein motor [126, 127]. Several *C. trachomatis* Incs (MrcA (myosin regulatory complex subunit A), CT222, IPAM, CT224, CT228, IncB, IncC, CT288, and CT850; **Table 1**) have been shown to concentrate at regions of the inclusion membrane near the centrosome, known as inclusion microdomains, which are also enriched in cholesterol and in the phosphorylated active form of Src family kinases [128]. These kinases are involved in the control of a wide range of cellular processes and they have been shown to play several roles in chlamydial infection of host cells that vary between *Chlamydia* species [129]. The stromal interaction molecule 1 (STIM1) is also present at inclusion microdomains [130], and has been localized to the ER-inclusion MCSs [131]. This suggested that inclusion microdomains [128], the ER-inclusion MCSs [120, 125], and the pathogen synapses [123], could correspond to the same structure [130], a possibility which needs to be further examined.

Based on the functions of the associated Incs, the inclusion microdomains have been described as platforms for interaction with the centrosome, microtubules and the actin-myosin cytoskeleton [128, 132]. As described above, IPAM binds a centrosomal protein (CEP170) to modulate the microtubule cytoskeleton [109]; in addition, the ectopic expression of IPAM or of Inc CT224 (as well as of Inc CT225, which, up to now, has not been shown to concentrate at inclusion microdomains) in uninfected mammalian cells inhibits cytokinesis [133]. Although little is known about the function of *C. trachomatis* IncB, its orthologue from *C. psittaci* can bind synaptosome-associated protein (SNAP)-associated protein (SNAPIN) and this might enable the inclusion to associate with microtubules [134]. Inc CT288 binds the centrosomal protein coiled-coil domain containing 146 (CCDC146) [135]; CCDC146 is recruited to the periphery of the inclusion but this is independent of CT288, which might however modulate the function of CCDC146 at the inclusion [135]. Inc CT850 binds dynein light chain Tctex-type 1 (DYNLT1) [132], a dynein subunit; DYNLT1 is found at inclusion microdomains and its depletion affects the association of the inclusion with centrosomes [132]. CT228 and MrcA control chlamydial host cell exit by extrusion and what is known about their function is described below. Finally, some Incs in the microdomains could be primarily involved in heterophilic Inc:Inc interactions helping to stabilize the inclusions and/or organize the microdomains [128], which is also described further below.

### Incs controlling chlamydial exit from infected host cells

Chlamydial release from infected cells can occur by host cell lysis (preceded by permeabilization of the inclusion membrane) or extrusion (in which the entire inclusion is ejected from the host cell) [105] **(Figure 1)**. *C. trachomatis* Inc proteins MrcA and CT228 **(Table 1)** have been shown to regulate extrusion.

In the first description of chlamydial extrusion, it was shown that this process is dependent on the actin motor protein non-muscle myosin II [105]. This motor is comprised by different subunits including a heavy chain with motor and contractile properties (e.g., myosin IIA or IIB isoforms) and a regulatory light chain (e.g., myosin light chain 2 (MLC2)) [136]. Phosphorylation of MLC2 by myosin light chain kinase (MLCK) strongly enhances the activity of the motor, which is inhibited when MLC2 is dephosphorylated by myosin phosphatase. In turn, the activities of MLCK and of the myosin phosphatase are also regulated. For example, the activity of MLCK requires activation by Ca^2+^/calmodulin [136], and the myosin phosphatase is inactive when one of its subunits, the myosin phosphatase target subunit 1 (MYPT1), is phosphorylated [137]. *C. trachomatis* infection of host cells depleted of MLCK, MLC2, or myosin IIA or IIB, showed that chlamydial extrusion depends on all these proteins [138].

Yeast two-hybrid screens revealed that MrcA binds the inositol 1,4,5-trisphosphate receptor type 3 (ITPR3) [130], belonging to a family of channels mediating release of Ca^2+^ from intracellular stores [139], and that Inc CT228 binds human MYPT1 [138]. The MrcA:ITPR3 and CT228:MYPT1 interactions were validated in *C. trachomatis*-infected cells [130, 138] **(Figure 3D)**. Infection of cultured cells using wild-type *C. trachomatis*, and *mrcA* or *ct228* null mutant strains, revealed that MrcA is necessary for the localization of ITPR3 at inclusion microdomains [130], and that CT228 is needed for the presence of MYPT1 at the periphery of the inclusion [140]. Furthermore, MrcA and ITPR3 are required for the localization of the phosphorylated forms of MYPT1 (inactive) and of MLC2 (active) at inclusion microdomains [130]. The phosphorylated active form of MLCK also localizes at inclusion microdomains but this is MrcA- and CT228-independent [130, 138, 140]. Finally, myosin IIA and IIB can also be detected at inclusion microdomains, but this is at least CT228-independent [138, 140]. Thus, MrcA recruits ITPR3 to inclusion microdomains, while CT228 recruits MYPT1 to the inclusion periphery. The localization at inclusion microdomains of the phosphorylated forms of MYPT1 and MLC2, both indicative of localized activity of myosin II, is also MrcA-dependent, likely through the MrcA:ITPR3 interaction **(Figure 3D)**.

Infection of cultured cells with wild-type or *mrcA* mutant strains revealed that extrusion is promoted by MrcA [130]. Furthermore, as mentioned above, STIM1 has been localized at inclusion microdomains and, as ITPR3, STIM1 also controls intracellular Ca^2+^ [130, 141]. In uninfected cells, STIM1 localizes at the ER where it senses depletion of Ca^2+^ stores and, in these conditions, mediates the influx of Ca^2+^ into cells [141]. Presumably there is an Inc, which remains to be identified, recruiting STIM1 to the inclusion membrane **(Figure 3D)**. Cultured cells infected by wild-type *C. trachomatis* after depletion of ITPR3 or STIM1, or after chelation of intracellular Ca^2+^, all showed a reduction in inclusion extrusion [130]. Therefore, Ca^2+^ signalling is modulated by *C. trachomatis* to promote inclusion extrusion, at least through the MrcA:ITPR3 interaction and by co-option/subversion of STIM1 [130]. Interference with Ca^2+^ signalling could help *C. trachomatis* to promote the activation of the Ca^2+^/calmodulin-dependent kinase MLCK or to activate kinases that phosphorylate/inhibit MYPT1 [130, 136]. Regardless of the exact mechanism, this would activate myosin II activity by favouring the phosphorylated active state of MLC2. On the other hand, infection of cultured cells with wild-type *C. trachomatis* or *ct228* null mutant strains showed that inclusion extrusion is inhibited by CT228 [140]. In addition, infection experiments in MYPT1-depleted cells indicate that this inhibitory role of CT228 depends on MYPT1 [140].

In summary, the direct or indirect action of Inc proteins on the enzymes (myosin phosphatase or MLCK) that control the phosphorylation state of MLC2 enables *C. trachomatis* to regulate inclusion extrusion by modulating myosin II activity. But why is this important for *C. trachomatis*? The *mrcA* mutant showed a slight growth defect in cultured cells that, however, could not be complemented [130]. On the other hand, the *ct228* mutant was not impaired for growth in cultured cells but showed a delay in clearance and a reduction in systemic humoral response in a mouse model of infection [140]. This has implications for *in vivo* infection, also suggested by studies indicating that extrusion facilitates the subsequent contact of *C. trachomatis* with dendritic cells and macrophages [142, 143].

### Incs binding 14-3-3 proteins

The 14-3-3 phospho-serine/phospho-threonine binding proteins are present in all eukaryotic cells, where they have many and varied interacting partners and regulate a wide diversity of cellular processes [144]. Different 14-3-3 protein isoforms have been shown to bind several Inc proteins. The 14-3-3β isoform was even the first host protein shown to bind an Inc (IncG) and to be recruited to the periphery of the inclusion membrane [145]. More recently, immunoprecipitation of Incs ectopically expressed in cultured cells followed by mass spectrometry revealed the binding of different 14-3-3 isoforms to InaC (ε, η, ζ, γ, θ, and β isoforms) [15] and to Inc CT006 (γ, β, and η isoforms) [85]. The interaction between InaC and 14-3-3β and 14-3-3ε was further validated, and the recruitment of these two 14-3-3 isoforms to the periphery of the inclusion membrane was shown to involve InaC [15]. There is evidence suggesting that 14-3-3β sequesters the BCL2 associated agonist of cell death (BAD) protein at the periphery of the inclusion membrane to protect the host cell from apoptosis [146]. However, in general, the role in *C. trachomatis* host cell infection of the 14-3-3 proteins and of its reported interactions with Inc proteins is unclear.

### Incs involved in Inc:Inc heterophilic interactions

Many Incs have relatively long stretches of their polypeptide chains predicted to be exposed on the host cell cytosol, while others have putative host cytosolic regions of less than 30 amino acid residues [147]. This suggested that Incs predictably less exposed to the host cell cytosol could be mainly involved in Inc:Inc interactions required for the stability of the inclusion or to organize the functions of other host protein-interacting Incs [147]. The latter hypothesis has also been suggested by analyses of the ectopic expression of Incs in cultured human cells [148]. Inc:Inc interactions have been directly screened by bacterial two-hybrid, and this suggested or confirmed several homotypic (IncV, IncD, IncF, IncA, CT222, IPAM, IncC, CT249, and InaC) and heterotypic Inc:Inc interactions [75]. In particular, IncF and CT222 should have small regions exposed to the host cell cytosol and they were shown to bind several other Incs [75]: IncF to IncV, IncD, IncG, IncC, IncA, CT249 and CT850, and CT222 to IncD, IPAM, CT224, and CT850. The CT222:CT850 interaction was also detected in *C. trachomatis*-infected cells [128]. Furthermore, an IPAM:CpoS interaction revealed by bacterial two-hybrid [75] was also detected in infected cells [98].

Several of the Incs involved in heterophilic Inc:Inc interactions have been localized to ER-inclusion MCSs (IncV, IncD) or to inclusion microdomains (CT222, IPAM, CT224, IncC, CT850). A *C. trachomatis incC* mutant revealed an unstable vacuolar membrane [100], consistent with the possible involvement of IncC in heterophilic Inc-Inc interactions; however, the disruption of *incV, ct224*, and *ct850* did not lead to unstable inclusions [100].

### An *inc* gene important for virulence *in vivo*, but inactivated during *in vitro* passage

Different lines of evidence indicate that *in vitro* serial passage of trachoma and genital *C. trachomatis* strains, but not of LGV strains, leads to the inactivation of the *ct135* gene [149–153], encoding an Inc **(Table 1)**. Because the *ct135* gene is intact in low-passage genital clinical isolates, this indicates that, at least in these strains, *ct135* is under positive selection *in vitro*, and negative selection *in vivo* [153]. Accordingly, a *C. trachomatis* serovar D strain with a single nucleotide insertion in the middle of the *ct135* gene (which might correspond to a null mutation) is less virulent in a mouse infection model than an isogenic strain with a single nucleotide deletion in the beginning of the *ct135* gene (which might enable the putative production of a near intact CT135 protein) [149, 150]. In contrast, these two strains do not display differences during their growth *in vitro* [149]. Therefore, although its mode of action is presently unknown, CT135 is important for *C. trachomatis* virulence *in vivo*.

## *C. TRACHOMATIS* NON-INC PROTEINS DELIVERED TO THE OUTSIDE OF THE INCLUSION

Besides Incs, 24 *C. trachomatis* proteins have been shown to be delivered outside of the inclusion and detected in the host cell plasma membrane, cytoplasm or nucleus, and/or at the inclusion membrane **(Table 2)**. Ten of these proteins have also been detected within the inclusion lumen, outside of the chlamydiae **(Table 2)**. Furthermore, although their delivery outside of the inclusion was never formally shown, there are *C. trachomatis* proteins (CT166, CT619, CT712 and CT849) for which there is evidence for an effector role **(see Table 2 footnote)**. We will not consider *C. trachomatis* secreted proteins that likely form the T3S system translocon either in the host cell plasma membrane or in the inclusion membrane (CT578/CTL0841/CopB, CT579/CTL0842/CopD, CT860/CTL0235/CopD2, CT861/CTL0236/CopB2) [63, 154, 155], or the T3S system needle at the bacterial surface (CT666/CTL0035/CdsF) [156], or that might control T3S system needle length (CT671/CTL0040/CdsP) [63, 157].

**TABLE 2:** *C. trachomatis* non-Inc proteins secreted into the host cell cytoplasm, inclusion membrane, or inclusion lumen^a^.

**Protein (annotation/name)**			**Secretion pathway; host cell protein targets; localization in infected host cells; and proposed function(s) and/or activity**	**References**
**Strain D/UW3**	**Strain L2/434**	**General**		
CT042	CTL0298	GlgX	*Secretion*: T3S; *targets*: N/A;* localization*: inclusion lumen and membrane; *function*: glycogen hydrolase.	[289]
CT049	CTL0305	Pls1	*Secretion*: unknown but T3S-independent; *targets*: unknown;* localization*: inclusion lumen; *function*: unknown.	[293]
CT050	CTL0306	Pls2	*Secretion*: unknown but T3S-independent; *targets*: unknown;* localization*: inclusion lumen; *function*: unknown.	[293]
CT089	CTL0344	CopN	*Secretion*: T3S; *targets*: unknown;* localization*: inclusion membrane; *function*: regulation of the T3S system.	[23, 50]
CT105	CTL0360	CteG	*Secretion*: T3S; *targets*: unknown; *localization*: Golgi and host cell plasma membrane; *function*: modulation of eukaryotic vesicular trafficking.	[227, 228]
CT142	CTL0397	-	*Secretion*: T3S; *targets*: unknown; *localization*: inclusion lumen; *function*: unknown.	[227, 294]
CT143	CTL0398	-	*Secretion*: T3S; *targets*: unknown; *localization*: inclusion lumen; *function*: possible involvement in inflammatory processes.	[227, 292, 294–296]
CT144	CTL0399	-	*Secretion*: T3S; *targets*: unknown; *localization*: inclusion lumen; *function*: unknown.	[227, 294, 295]
CT156	Absent	Lda1	*Secretion*: unknown; *targets*: unknown; *localization*: around the inclusion, overlapping with LD-like structures; *function*: targeting LDs.	[54, 219]
CT163	CTL0419	Lda2	*Secretion*: unknown; *targets*: unknown; *localization*: around the inclusion, overlapping with LD-like structures, and within LD biochemical fractions; *function*: targeting LDs.	[54, 219]
CT311	CTL0563	-	*Secretion*: Sec-dependent/T2S; *targets*: unknown; *localization*: inclusion lumen, cytosol and nucleus; *function*: unknown.	[32, 279]
CT456	CTL0716	TarP	*Secretion*: T3S; *targets*: actin, ABI1, VAV2, PI3K p85 subunit, SHC1; *localization*: cytosol, near the nascent inclusion, and membrane fractions of infected cells; *function*: actin nucleator; modulation of actin-mediated changes involved in host cell invasion, and of host cell survival.	[158, 170, 175–178, 186, 190]
CT473	CTL0734	Lda3	*Secretion*: unknown; *targets*: unknown; *localization*: around the inclusion, overlapping with LD-like structures; *function*: targeting and modulation of LDs.	[54, 219, 220]
CT529	CTL0791	Cap1	*Secretion*: T3S; *targets*: unknown; *localization*: inclusion membrane; *function*: associates with LDs.	[50, 63, 221, 280]
CT620	CTL0884	-	*Secretion*: T3S; *targets*: Hrs/ESCRT machinery; *localization*: inclusion lumen, cytosol and nucleus; *function*: unknown.	[212, 214]
CT621	CTL0885	-	*Secretion*: T3S; *targets*: Hrs/ESCRT machinery; *localization*: inclusion lumen, cytosol and nucleus; *function*: unknown.	[212–214]
CT622	CTL0886	-	*Secretion*: T3S; *targets*: unknown; *localization*: inclusion lumen and cytosol; *function*: involved in bacterial infectivity and growth; structural similarities with GGTases and synthases.	[200, 201]
CT694	CTL0063	TmeA	*Secretion*: T3S; *targets*: AHNAK; *localization*: cytosol, near the nascent inclusion, and host cell plasma membrane; *function*: involved in host cell invasion.	[185, 186, 189–191]
CT695	CTL0064	TmeB	*Secretion*: T3S; *targets*: unknown; *localization*: cytosol, near the nascent inclusion, and inclusion membrane; *function*: unknown.	[185, 186]
CT711	CTL0080	-	*Secretion*: T3S; *targets*: Hrs/ESCRT machinery; *localization*: nucleus; *function*: unknown.	[212, 214]
CT737	CTL0106	NUE	*Secretion*: T3S; *targets*: histones H2B, H3 and H4; *localization*: nucleus; *function*: histone methyltransferase.	[226]
CT795	CTL0164	-	*Secretion*: Sec-dependent/T2S; *targets*: unknown; *localization*: inclusion lumen and cytosol; *function*: unknown.	[278]
CT798	CTL0167	GlgA	*Secretion*: T3S; *targets*: N/A; *localization*: inclusion lumen and cytosol; *function*: glycogen synthase.	[288]
CT806	CTL0175	Ptr	*Secretion*: Sec-dependent/T2S; *targets*: N/A; *localization*: inclusion lumen; *function*: putative protease involved in recovery of *C. trachomatis* from IFN?-induced stress.	[292]
CT823	CTL0195	HtrA	*Secretion*: Sec-dependent/T2S; *targets*: unknown; *localization*: inclusion lumen and cytosol; *function*: serine protease with roles in the chlamydial periplasm, but unknown function in the host cell cytosol.	[261, 262]
CT858	CTL0233	CPAF	*Secretion*: Sec-dependent/T2S; *targets*: several, but many shown to be experimental artifacts; *localization*: inclusion lumen and cytosol; *function*: promotion of chlamydial survival in the mouse lower genital tract; evasion of innate immune responses; chlamydial lytic exit; inhibition of host cell cytokinesis; inhibition of p65 nuclear translocation (based on phenotypes displayed by *cpaf* mutant *C. trachomatis*).	[231, 232, 234–237, 240, 241, 245, 248, 250–252, 255]
CT867	CTL0246	ChlaDUB2 /Cdu2	*Secretion*: unknown; *targets*: unknown; *localization*: inclusion membrane and host cell plasma membrane; *function*: DUB and deneddylase activities; contributes to Golgi redistribution around the inclusion.	[189, 204, 206]
CT868	CTL0247	ChlaDUB1/ Cdu1	*Secretion*: unknown; *targets*: MCL1, I?B?; *localization*: inclusion membrane; *function*: DUB, deneddylase and acetyl transferase activities; contributes to Golgi redistribution around the inclusion; inhibition of NF-?B signalling and of host cell death.	[11, 189, 204, 206, 209]
CT875	CTL0255	TepP	*Secretion*: T3S; *targets*: CRK, CRKL, PI3K subunits, and GSK3B; *localization*: cytosol, near the early vacuole; *function*: modulation of host gene expression related with immune signalling in early stages of infection.	[195, 196]
pGP3/pORF5			*Secretion*: unknown; *targets*: cathelicidin LL-37 (antimicrobial peptide); *localization*: inclusion lumen and cytosol; *function*: neutralization of the LL-37activity, evasion of the acidic barrier in the vagina, modulation of inflammatory responses, inhibition of host cell apoptosis.	[268, 274–277]

a Only *C. trachomatis* proteins reported to be experimentally detected in the inclusion membrane, inclusion lumen, host cell cytoplasm or nucleus are listed. In addition, there is functional evidence for secretion and effector role of CT166 (*Chlamydia* cytotoxin targeting RAC1 that might contribute for downmodulating actin cytoskeleton changes during chlamydial invasion of host cells [197, 199]), CT619/CTL0883 and CT712/CTL0081 (as CT620/CTL0884, CT621/CTL0885, and CT711/CTL0080, listed in Table 2, these two proteins contain the *Chlamydiacea*-unique DUF582 domain and bind components of the ESCRT machinery [212, 214]), and CT847/CTL0217 (binds GCIP [229]) in the host cell cytoplasm, and of GlgB, GlgP, MalQ and MrsA in the inclusion lumen [14, 289]. See list of abbreviations and main text for abbreviations and protein nomenclature, respectively.

### Effector proteins packed in EBs

**Figure 4:**
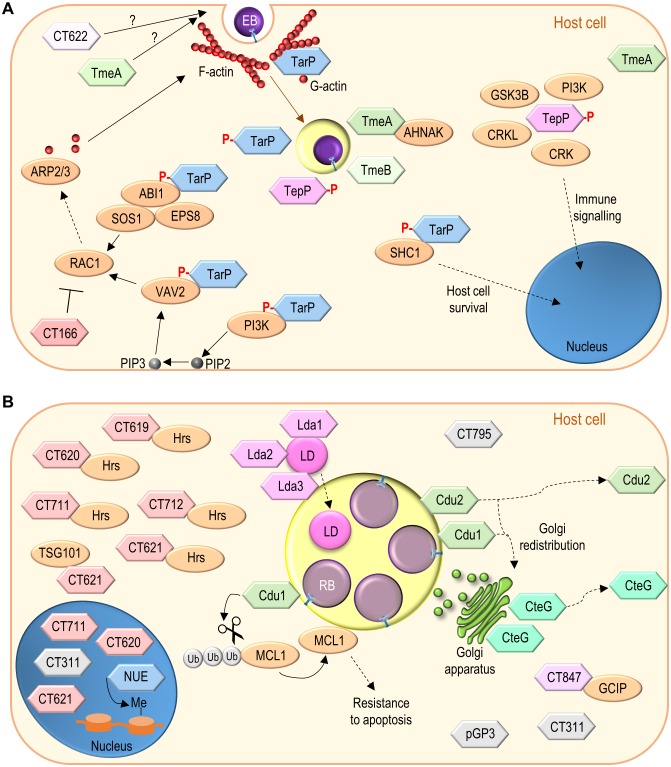
FIGURE 4: Function of non-Inc *C. trachomatis* effectors. **(A)** *C. trachomatis* effectors (TarP, TmeA, TmeB, CT166, CT622) packed in EBs and delivered into host cells during invasion; besides TarP, TmeA and CT622 also promote *C. trachomatis* invasion but their mode of action is unknown. **(B)** *C. trachomatis* non-Inc proteins that have been detected in the host cell cytoplasm, nucleus, or inclusion membrane (Cdu1, Cdu2, CteG, Lda1, Lda2, Lda3, NUE, pGP3, CT311, CT620, CT621, CT711, and CT795; GlgX, CopN, Cap1, GlgA, HtrA, and CPAF are not represented) or for which there is functional evidence for an effector role (CT619, CT712, CT847). It is unclear whether these proteins are delivered into host cells by EBs or by RBs (as represented), or by both chlamydial forms. Among the chlamydial proteins represented and not binding a host cell protein, pGP3 has been shown to interact with the antimicrobial peptide cathelicidin LL-37 but this likely occurs extracellularly [276]. See list of abbreviations, main text, and **Table 2** for details.

The invasion of host cells by EBs involves first interactions between different chlamydial adhesin molecules and diverse eukaryotic receptors [6]. Furthermore, multiple evidence indicates that *C. trachomatis* EBs contain functional T3S systems and are packed with effector proteins injected upon contact of the chlamydiae with host cells [26, 158–161]. Some of these effectors have been identified and characterized; they at least modulate post-adhesion chlamydial invasion and interactions of the nascent inclusion with host cells **(Figure 4A)**.

#### The identification of TarP and characterization of its host cell targets and functions

Invasion of host cells by *C. trachomatis* induces protein tyrosine phosphorylation [162, 163], and involves alterations in the host cell actin cytoskeleton [104, 164]. The alterations in the actin cytoskeleton are dependent on the Rac family small GTPase 1 (RAC1) [165], and on the cell division cycle 42 (CDC42) protein [164], both belonging to the Rho family of small GTPases, involved in the control of the dynamics of microfilaments [166]. During *C. trachomatis* invasion, RAC1 recruits Wiskott-Aldrich syndrome protein family member 2 (WAVE2) and abl interactor 1 (ABI1), while CDC42 recruits Neural Wiskott-Aldrich syndrome protein (N-WASP), promoting the initiation of actin polymerization by the actin-related protein 2/3 (ARP2/3) complex [164, 167]. The *tour de force* that led to the identification of TarP (chlamydial translocated actin-recruiting phosphoprotein) **(Table 2)** was crucial for the ongoing clarification of how *Chlamydia* modulate all these processes [158].

Immunoprecipitation with an anti-phosphotyrosine antibody followed by mass spectrometry from protein extracts of cells infected by *C. trachomatis* revealed a tyrosine phosphorylated chlamydial protein [158]. Because the protein was associated with EBs but exposed in the host cell cytosol, it was hypothesized that it could be a T3S substrate, which was confirmed using *Yersinia* as heterologous bacteria [158]. Furthermore, actin was recruited to sites of tyrosine phosphorylation near EBs invading host cells, and ectopic expression in mammalian cells of the identified chlamydial protein also led to its tyrosine phosphorylation and to F-actin recruitment [158]. This indicated that *C. trachomatis* EBs use their T3S system to deliver an effector (TarP) into host cells that is then tyrosine phosphorylated, and which is associated with actin polymerization at chlamydial entry sites **(Figure 4A)**.

TarP is present in all *C. trachomatis* serovars and *Chlamydia* species [168, 169]. *C. trachomatis* TarP includes a tyrosine-rich repeat domain corresponding to the region that is tyrosine phosphorylated [168]. The number of tyrosine-rich repeats is variable among TarP from different *C. trachomatis* serovars and is absent in TarP from other *Chlamydia* species [168–170]. Notwithstanding, *C. trachomatis* TarP is phosphorylated by Abl and Src family tyrosine kinases [171, 172], and possibly by other kinases [173]. In eukaryotic cells, tyrosine phosphorylated proteins are often recognized by SRC homology 2 (SH2) domains in other proteins [174]. In the case of TarP, its tyrosine phosphorylation within host cells has been shown to mediate binding to the SH2 domain-containing adaptor protein 1 (SHC1) and subsequent activation of signalling involving mitogen-activated protein kinases that promotes host cell survival [175] **(Figure 4A)**.

The tyrosine-rich repeats of *C. trachomatis* TarP are also associated with binding to host cell proteins which mediates signalling leading to actin polymerization [176]. Ectopic expression in mammalian cells of a hybrid protein comprising only one of the tyrosine-rich repeats of *C. trachomatis* TarP leads to its tyrosine phosphorylation and to F-actin and RAC1 recruitment [176]. Furthermore, an oligopeptide with a single *C. trachomatis* tyrosine-rich repeat carrying phosphorylated tyrosines pulls down RAC1 and its activators, the SH2 domain-containing vav guanine nucleotide exchange factor 2 (VAV2), and a complex formed by ABI1, SOS Ras/Rac guanine nucleotide exchange factor 1 (SOS1), and epidermal growth factor receptor pathway substrate 8 (EPS8) [176]. The binding of TarP to the ABI1:SOS1:EPS8 complex is likely mediated by ABI1 and is probably indirect, because ABI1 lacks a SH2 domain [176]. The activation of RAC1 by VAV2 requires the phospholipid phosphatidylinositol (3,4,5)-trisphosphate (PIP3), which is present at chlamydial entry sites [176]. Accordingly, the SH2 domain-containing p85 subunit of a class I phosphatidylinositol 3-kinase (PI3K), which generates PIP3 from plasma membrane-abundant phosphatidylinositol (4,5)-bisphosphate (PIP2), also binds to a TarP-derived oligopeptide with phosphorylated tyrosines [176]. VAV2, SOS1, ABI1, and EPS8 are recruited and colocalize at sites of *C. trachomatis* entry into host cells, and are all required for chlamydial invasion [176]. In agreement with a role of TarP phosphorylation in *C. trachomatis* invasion, the chlamydial uptake process is inhibited after expression in *C. trachomatis* of a TarP mutant protein lacking the tyrosine-rich repeats [177]. This dominant-negative effect is a consequence of TarP oligomerization through a proline-rich domain [177, 178]. Overall, this suggests a model in which tyrosine phosphorylated *C. trachomatis* TarP binds and recruits RAC1 activators (VAV2 and the ABI1:SOS1:EPS8 complex), ultimately leading (through WASP2 and ABI1) to the initiation of localized actin polymerization by the ARP2/3 complex and promotion of chlamydial invasion of host cells [176] **(Figure 4A)**.

TarP proteins lacking tyrosine-rich repeats also recruit F-actin, indicating that tyrosine phosphorylation of TarP is not an absolute requirement for actin recruitment and host cell invasion [168]. In fact, TarP also possesses globular (G)-actin binding and F-actin-binding domains that are present in all *C. trachomatis* serovars and *Chlamydia* species [169, 178] **(Figure 4A)**. TarP binds actin directly, promotes its nucleation *in vitro* [178], and can cooperate with the ARP2/3 complex to increase the rate of actin polymerization *in vitro* [179]. The F-actin-binding domains of TarP mediate bundling of actin filaments, an activity that does not require the G-actin binding domain [180]. TarP mutant proteins lacking the G-actin-binding domain inhibit actin polymerization by the wild-type protein and when expressed in *C. trachomatis* they reduce the ability of the recombinant chlamydiae to invade host cells [177]. Similarly, an antibody specifically recognizing the G-actin-binding domain of TarP inhibits both actin polymerization *in vitro* and chlamydial entry into host cells [170]. Overall, this indicates that TarP and its actin-binding domains are important for *C. trachomatis* invasion.

In summary, delivery of TarP into host cells by the T3S system in *C. trachomatis* EBs leads to the tyrosine phosphorylation of the effector within infected cells. This activates signalling pathways likely promoting host cell survival and localized actin polymerization resulting in chlamydial invasion **(Figure 4A)**. Direct actin nucleation and bundling by TarP should also mediate *C. trachomatis* invasion. Furthermore, *C. caviae* TarP has been shown to target focal adhesion kinase (FAK) and vinculin during chlamydial invasion through protein motifs also present in TarP orthologues from other *Chlamydia* species, including *C. trachomatis* [181, 182]. Finally, the *C. pneumoniae* TarP orthologue, which also binds vinculin [183], can stabilize F-actin by preventing the activity of the host actin-severing protein cofilin [184].

#### The membrane associated TmeA and TmeB: chlamydial invasion and more

The idea that chlamydial genes with mRNA levels first detected late in the chlamydial developmental cycle could be candidates for encoding proteins packed in EBs led to the identification of *C. trachomatis* TmeA (translocated membrane-associated effector A) and TmeB **(Table 2)** as T3S effectors [185, 186].

TmeA and TmeB are encoded by a small *tmeAB* operon [186]. They are indeed present in *C. trachomatis* EBs [159, 185, 186], and their secretion by *Yersinia* is T3S-dependent [185, 187]. IF microscopy of *C. trachomatis*-infected cells revealed that TmeA and TmeB localize in the host cell cytoplasm, nearby the nascent inclusion, between 1-3 h post-infection [185, 186] **(Figure 4A)**. The TEM-1 β-lactamase reporter assay, enabling to monitor the delivery of bacterial effector proteins into mammalian host cells [188], allowed to detect TmeA and TmeB (and TarP) in the host cell cytoplasm 24 h post-infection [186]. Furthermore, at 24 h post-infection, TmeA was detected at the host cell plasma membrane by using the split-GFP technology [189], and TmeB was detected around the inclusion membrane by IF microscopy [186]. Biochemical experiments also showed the association of TmeA with membranes in *C. trachomatis*-infected cells [190]. In summary, TmeA and TmeB are delivered by *C. trachomatis* into host cells early in infection and they localize near the nascent inclusion. Later in infection, TmeA associates with the host cell plasma membrane and TmeB remains associated with the inclusion membrane.

*C. trachomatis tmeA* and *tmeB* mutants have been generated [10, 12]. Characterization of the mutant strains revealed defects of the *tmeA* mutants for invasion of host cells and in a mouse infection model [12, 191], but thus far no defects have been reported for the *tmeB* mutant [191]. The mechanism by which TmeA promotes chlamydial invasion is presently unknown. A yeast two-hybrid screen revealed that TmeA binds human AHNAK nucleoprotein (AHNAK) [185]; this interaction was also detected in mammalian cells infected by *C. trachomatis* for 5 h [191] **(Figure 4A)**. AHNAK binds actin and mediates F-actin bundling [192, 193], and TmeA can inhibit this actin bundling activity of AHNAK *in vitro* [191]. However, transient recruitment of endogenous AHNAK to the nascent inclusion is independent of TmeA [191]. Moreover, a *C. trachomatis tmeA* mutant shows a defect in host cell invasion regardless of the cells being AHNAK-positive or AHNAK knocked-out, and there is no defect in invasion associated with infection of AHNAK knocked-out cells by wild-type *C. trachomatis* [191]. Therefore, TmeA is required for efficient invasion of host cells by *C. trachomatis* but this is apparently independent of the TmeA:AHNAK interaction.

#### Modulation of immune signalling by TepP

Many T3S effectors require specific bacterial cytosolic chaperones for their proper delivery into target host cells [20]. TarP, TmeA and TmeB had been shown to share the same T3S chaperone (Slc1; SycE-like chaperone 1) [187, 194]. Additional binding partners of Slc1 were then searched for within *C. trachomatis* EBs through immunoprecipitation of Slc1 followed by mass spectrometry analysis of pulled down proteins [195]. This led to the identification of a *C. trachomatis* T3S effector protein that based on its properties was named TepP (translocated early phosphoprotein) **(Table 2)**.

TepP is secreted by *Yersinia* in a T3S-dependent manner, which is promoted by Slc1 [195], as also observed for other Slc1 partners (TarP, TmeA and TmeB) [187, 194]. As TarP, TmeA, and TmeB (see above), TepP is delivered into the cytoplasm of *C. trachomatis* infected cells and localizes near the nascent inclusion [195] **(Figure 4A)**. Furthermore, as TarP, TepP is rapidly tyrosine phosphorylated by host cell Src family kinases after its chlamydiae-mediated delivery into infected cells [195, 196]. However, TepP is also phosphorylated at serine residues [195].

Two different *tepP* mutant strains have been isolated and characterized [195, 196]. *C. trachomatis* strains carrying nonsense or inactivating insertion mutations in *tepP* did not show defects in host cell invasion or in chlamydial growth in HeLa cells [195, 196], but the *tepP* insertional mutant strain was defective for growth in A2EN cervical epithelial cells [196]. Furthermore, A2EN cells infected by both *tepP* mutant strains showed alterations in the expression of genes associated with innate immune responses, including type I IFN responses, such as reduced induction of IFN-induced peptides with the tetratricopeptide repeat (*IFIT*) genes [195, 196].

In *C. trachomatis*-infected cells, TepP has been shown to bind the CRK proto-oncogene, adaptor protein (CRK), CRK like proto-oncogene, adaptor protein (CRKL), glycogen synthase kinase 3β (GSK3B), and different subunits of class I PI3K [195, 196] **(Figure 4A)**. CRK, CRKL, and PI3K (and GSK3B, but to a lesser extent) are recruited to the proximity of the nascent inclusion in a TepP-dependent manner [195, 196]. Binding to, and recruitment of, at least CRKL and PI3K does not depend on tyrosine phosphorylation of TepP by Src family kinases [196]. However, the induction in the expression of *IFIT* genes in cells infected by TepP-expressing chlamydiae depends on PI3K [196]. This could be related with increased PI3K activity in the vicinity of the nascent inclusion by TepP-recruited PI3K [196].

In summary, TepP is a *C. trachomatis* T3S effector recruiting CRK and CRKL adaptor proteins, as well as PI3K, to modulate innate immune signalling early in host cell infection that is likely required for chlamydial growth.

#### Disruption of the host cell actin cytoskeleton by the C. trachomatis cytotoxin

Chlamydial genomes encode proteins displaying homology with the large *Clostridium difficile* toxins [197], which inactivate Rho family GTPases through their glucosyltransferase activity [198]. Some of these chlamydial proteins show conservation of the amino acid residues critical for the glucosyltransferase activity of large clostridial toxins [197]. This is the case of CT166 **(Table 2)**, encoded by some, but not all, *C. trachomatis* strains**.** The CT166-encoding strains cause a cytopathic effect (cell rounding and dramatic alterations in the actin cytoskeleton) in cultured cells, and the severity of the effect correlates with the multiplicity of infection [197]. Ectopic expression of CT166 in mammalian cells recapitulates the cytopathic effect observed in C*. trachomatis*-infected cells [199]. This effect of ectopically expressed CT166 depends on RAC1 and on the conserved amino acid residues required for glucosyltransferase activity of the clostridial toxins [199]. The CT166 protein is present in EBs and can be detected in protein extracts of cells infected by *C. trachomatis* up to 1 h post-infection [197]. Furthermore, in infected cells, the cytopathic effect does not require chlamydial transcription and translation [197].

In summary, while direct evidence is lacking, CT166 is probably delivered into host cells by EBs through the T3S system of some *C. trachomatis* strains. Within the cytoplasm of infected cells, CT166 likely glucosylates and inactivates RAC1. This can potentially downmodulate the RAC1-mediated signalling resulting in actin cytoskeleton changes required for chlamydial invasion **(Figure 4A)**.

#### Disruption of the gene encoding the effector protein CT622 results in pleiotropic defects

The *C. trachomatis* CT622 protein was initially identified in the host cell cytosol and within the inclusion lumen [200]. The N-terminal region of CT622 can direct secretion of a hybrid protein by *S. flexneri* in a T3S-dependent manner [201]. Although CT622 has been detected in the host cell cytosol only from 36 h post-infection [200, 201], its presence within EBs bound to a possible T3S chaperone [159, 201], and the phenotypes associated with the *ct622* mutant strain (see below) [201], indicate a possible much earlier function in the host cell cytoplasm **(Figure 4A)**.

The 3D structure of the C-terminal of CT622 has been determined by X-ray crystallography and revealed similarity with geranylgeranyl transferases (GGTases) and synthases [201]. Proteins with the GGTase activity transfer a 20-carbon lipophilic chain (geranylgeranyl) to the C-terminus of its specific targets [202], which, is for example, essential for the association of RAB proteins with cellular membranes. However, up to now, *in vitro* GGTase activity has not been detected for CT622 [201]. On the other hand, characterization of a *C. trachomatis ct622* mutant revealed several defects during the chlamydial developmental cycle [201]. The mutant strain shows reduced production of infectious progeny that is at least, but not only, related to defects in chlamydial invasion of host cells and in EB to RB conversion [201]. Furthermore, the initial *C. trachomatis*-dependent protein tyrosine phosphorylation is much reduced after infection by the *ct622* mutant strain [201]. Overall, this indicates that CT622 is an effector protein important throughout the developmental cycle of *C. trachomatis*.

### *C. trachomatis* deubiquitinases

The reversible post-translational modification of proteins by ubiquitination, or by other ubiquitin-like modifications like neddylation, is fundamental to control several eukaryotic cell processes and is often targeted by pathogens [203]. In the case of *C. trachomatis*, a ubiquitin-based probe was used to search for possible deubiquitinases (DUBs) within infected cells [204]. This eventually led to the identification of two chlamydial DUBs, named Cdu1 (also known as ChlaDUB1) and Cdu2 (also known as ChlaDUB2) [11, 204, 205] **(Table 2)**. Cdu1 or Cdu2 display DUB and deneddylase activities when ectopically expressed in mammalian cells [204]. Furthermore, purified Cdu1 has both DUB and acetyltransferase activities [206]. This *in vitro* DUB and acetyltransferase activities of Cdu1 involve the same catalytic centre in the enzyme, and the dual specificity is conferred by a helix that can contact either ubiquitin or coenzyme A [206].

In *C. trachomatis*-infected cells, Cdu1 and Cdu2 were shown to be delivered into the cytoplasm of host cells and, at 24 h post-infection, they were both detected at the inclusion membrane [11, 189] **(Figure 4B)**. However, at 48 h post-infection, while Cdu1 localizes only at the inclusion membrane, Cdu2 is also detected at the host cell plasma membrane [189] **(Figure 4B)**. Both chlamydial DUBs possess a transmembrane helix within their N-terminal region that might mediate insertion into cellular membranes. Although the mechanism by which Cdu1 and Cdu2 are delivered by the chlamydiae into the cytoplasm of host cells has not been directly addressed, bioinformatics strongly suggests that they are T3S substrates [207].

*C. trachomatis cdu1* and *cdu2* null-mutants have been isolated and characterized [11, 206]. By comparison to the wild-type strain, a *C. trachomatis cdu1* null-mutant showed reduced generation of infectious progeny in A549 cells (a lung epithelial cell line) [206], or in IFNγ-stimulated primary human fimbriae cells [11], but not in HeLa cells [206]. Furthermore, the *cdu1* mutant strain displayed a defect in a mouse model of infection [11]. In contrast, a *cdu2* null-mutant strain did not show a defect in the generation of infectious progeny in both A549 and HeLa cells [206]. Regarding *C. trachomatis*-induced host cell phenotypes, the characteristic Golgi redistribution around the inclusion was not observed in cells infected by *cdu1* or *cdu2* null mutants [206]. Accordingly, ectopic expression of Cdu1 or Cdu2 in mammalian cells induces Golgi fragmentation; for Cdu1 this is correlated with its DUB activity but not with its acetyltransferase activity [206]. This indicates that, as *C. trachomatis* InaC (see above), Cdu1 and Cdu2 are involved in Golgi redistribution during chlamydial infection **(Figure 4B)**.

The nuclear factor-κB (NF-κB) family of transcription factors controls several mammalian genes with important roles in immunity [208]. Ectopically expressed Cdu1 suppresses NF-κB activation and binds the NF-κB inhibitor α (IκBα) [209], but it remains unknown whether this is relevant during infection. In addition, Cdu1 binds the host cell protein MCL1 apoptosis regulator, BCL2 family member (MCL1) in *C. trachomatis* infected cells [11] **(Figure 4B)**. MCL1 is an anti-apoptotic protein [210] involved in the resistance of *Chlamydia*-infected cells to apoptosis [211]. In *C. trachomatis*-infected cells, MCL1 is stabilized by deubiquitination, preventing its degradation in the proteasome [11]. In fact, Cdu1 deubiquitinates MCL1 [11] **(Figure 4B)**. Moreover, in cells infected by wild-type *C. trachomatis*, deubiquitinated MCL1 accumulates around the inclusion, while in cells infected by the *cdu1* mutant there is an increase in ubiquitinated MCL1 at the inclusion and the overall cellular levels of MCL1 are decreased [11]. Still, cells infected by the *cdu1* mutant are not more sensitive to apoptosis than cells infected by the wild-type strain [11]. This could be related to compensatory survival signalling pathways that are increased in *cdu1*-infected cells [11].

In summary, *C. trachomatis* delivers two DUBs into infected host cells and both enzymes contribute to the characteristic Golgi redistribution around the inclusion. Furthermore, Cdu1 exerts its deubiquitinating activity at the inclusion membrane, helping to stabilize a host cell protein (MCL1) important for apoptosis-resistance in *Chlamydia*-infected cells.

### DUF582-containing *C. trachomatis* effectors

Each chlamydial genome encodes several proteins possessing a *Chlamydiacea*-specific domain of unknown function (DUF582) within their primary structure. In *C. trachomatis*, these DUF582-containing proteins are CT619, CT620, CT621, CT711, and CT712 **(Table 2)**. Although this has not been directly shown for all of them, it is generally assumed that the chlamydial DUF582-containing proteins are T3S substrates delivered into the cytoplasm of infected cells and that at least some of the DUF582 proteins are also transported into the host cell nucleus. This general concept derives from several experiments. First, DUF582-containing proteins from *C. trachomatis, C. pneumoniae* and *C. caviae* are T3S substrates, as deduced from experiments using *S. flexneri* as heterologous bacteria [63, 212]. Second, *C. trachomatis* DUF582-containing proteins have been detected in the cytoplasm (CT620, CT621) and nucleus (CT620, CT621, CT711) of infected cells [212, 213] **(Figure 4B)**. Finally, CT620, CT621, and CT711 also localize in the host cell nucleus after their ectopic expression in uninfected human cells [212].

At a functional level, *C. trachomatis* DUF582-containing proteins have been shown to bind components of the endosomal sorting complexes required for transport (ESCRT) machinery of host cells [214], mostly known for being involved in the formation of multivesicular bodies (MVBs) in the endolysosomal pathway [215]. The interaction between *C. trachomatis* DUF582 proteins and a component of ESCRT complexes (Hrs) was initially found in yeast two-hybrid screens using the C-terminal region of CT619 (containing the DUF582) as bait [214]. Subsequently, by yeast two-hybrid, the C-terminal DUF582-containing regions of CT619, CT711, and CT712 were shown, or confirmed, to bind Hrs, and the N-terminal region of CT619 was shown to bind another component of ESCRT complexes (tumor susceptibility 101 (TSG101)) [214]. Furthermore, co-IP experiments with ectopically expressed proteins indicated an interaction between each of the *C. trachomatis* DUF582 proteins and Hrs, and between CT619 and TSG101 [214]. Thus, *C. trachomatis* CT619, CT620, CT621, CT711, and CT712 can bind Hrs through their C-terminal DUF582 region, and CT619 can also bind TSG101 through its N-terminal region. The physiological significance of these interactions is presently unclear, as siRNA-mediated depletion of Hrs or TSG101 does not appear to interfere with *C. trachomatis* internalization or with the chlamydial developmental cycle [214]. Nevertheless, the interaction of DUF582-containing *C. trachomatis* proteins with components of the ESCRT machinery suggests that these chlamydial effectors could modulate the host cell endocytic pathway or other ESCRT-dependent processes. For example, the ESCRT machinery is also needed for host cell scission events, such as abscission during cytokinesis [215], and it has recently been shown to have a role in *C. trachomatis* exit by extrusion [216]. It is, however, currently unknown whether *C. trachomatis* DUF582-containing effectors regulate chlamydial extrusion.

### Lipid droplet-associated *C. trachomatis* proteins

Lipid droplets (LDs) are organelles playing an important role in lipid and energy homeostasis of eukaryotic cells [217]. They are also recognized as modulators of immune responses and are targeted by diverse pathogens [218]. In the case of *C. trachomatis*, the presence of LDs around and within the inclusion [219, 220], and the altered amounts of host cell lipids and proteins in LDs from *C. trachomatis-*infected cells [219, 221], indicates that these organelles are targeted by *Chlamydia*. The relevance of LDs for chlamydial infections is further supported by their detection within inclusions from cells of mice that had been infected by the mouse and hamster pathogen *C. muridarum* [222]. However, analyses of *C. trachomatis* growth in cells lacking LDs have produced conflicting data [221, 223, 224]. Furthermore, chlamydial growth defects observed in cells devoid of LDs [219, 223], appear to relate to reduced activity of long-chain acyl-CoA synthases (ACSLs), which are found within the inclusion either in the presence or absence of LDs [223].

Regardless of the role of LDs in chlamydial infections, several *C. trachomatis* proteins have been shown to associate with these organelles. Phenotypic and subcellular localization screens of a large collection of yeast strains expressing *C. trachomatis* proteins revealed four chlamydial LD-associated (Lda) proteins [54, 219]: Lda1, 2, and 3 (**Table 2**), and also CT257/CTL0509/Lda4, which has not been further studied. Ectopically expressed Lda1, 2 and 3 also associate with LDs in mammalian cells [219]. In *C. trachomatis*-infected cells, Lda1, 2, and 3 co-localize with LD-like structures surrounding the inclusion [219], and ectopically expressed Lda3 could also be found at the inclusion membrane and lumen [220]. Overall, this suggested a model in which cytoplasmic LDs, associated with Lda3, bind to an unknown Inc and this promotes translocation of LDs into the lumen of the inclusion [220]. Although the data is not consistent between the different studies where this was examined, Incs (IncA, IncG and CT618) [220, 221], the inclusion membrane localized Cap1 (class I accessible protein-1) [221], and Lda2 [219] have been found associated with LDs isolated from *C. trachomatis*-infected cells, providing some support to the model. Furthermore, ectopically expressed CT618 and Cap1 also associate with LDs in mammalian cells [221].

### A *C. trachomatis* nuclear effector that methylates histones

The first *C. trachomatis* genome revealed a gene potentially encoding a protein containing a SET (Su(var)3-9, Enhancer-of-zeste and Trithorax) domain [21], mostly found in eukaryotic histone methyltransferases controlling gene expression and chromatin structure [225]. The *C. trachomatis* SET domain-containing protein was deduced to be a T3S substrate based on secretion assays using *S. flexneri* as heterologous bacteria [226]. After biochemical fractionation of cells infected by *C. trachomatis,* the SET domain-containing chlamydial protein was found in the host cell nucleus associated with chromatin [226]. The chlamydial protein, named nuclear effector (NUE), also localized in the nucleus upon ectopic expression in mammalian cells [226]. Furthermore, in an *in vitro* system, NUE was capable of methylating histones H2B, H3 and H4 [226]. This showed that NUE is a *C. trachomatis* effector localizing in the nucleus of infected cells where it presumably methylates histones and therefore possibly remodels chromatin **(Figure 4B)**.

### From the chlamydiae to the Golgi complex and host cell plasma membrane

A screen for *C. trachomatis* T3S substrates using *Yersinia* as heterologous bacteria identified several candidate chlamydial effectors [227]. Additional studies showed that one of these candidates is delivered into the cytoplasm of infected cells, where it initially associates with the Golgi complex [228]. The protein was named CteG (*C. trachomatis* effector associated with the Golgi) [228] **(Figure 4B)**. However, as chlamydial infection of cultured cells advances in time, CteG is found progressively more associated with the host cell plasma membrane [228]. A *C. trachomatis cteG* insertional mutant was generated, but it did not show a chlamydial growth defect in cultured cells [228]. Furthermore, cells infected by the *cteG* mutant did not display an alteration in Golgi redistribution around the inclusion [228]. However, CteG interferes with eukaryotic vesicular trafficking when ectopically expressed in yeast [228]. In summary, CteG is a *C. trachomatis* effector with dual and sequential localization in infected cells, first associated with the Golgi and then with the host cell plasma membrane **(Figure 4B)**. CteG might interfere with host cell vesicular trafficking, but this remains to be shown in infected mammalian cells.

### A *C. trachomatis* effector that might modulate host cell proliferation

*C. trachomatis* protein CT847 was identified as a T3S substrate using *Yersinia* as heterologous bacteria [229]. A yeast two-hybrid screen revealed that CT847 binds human Grap2 cyclin D-interacting protein (GCIP) [229], which contains a helix-loop-helix and might normally function as a transcription regulator controlling cell proliferation [230]. The interaction between CT847 and GCIP was validated in *C. trachomatis*-infected cells ectopically expressing GCIP [229] **(Figure 4B)**. Infection of mammalian cells by *C. trachomatis* leads to a depletion in the cellular levels of GCIP that is prevented by inhibitors of bacterial protein synthesis, of the T3S system, or of the host cell proteasome [229]. Accordingly, siRNA-mediated depletion of GCIP leads to increased production of *C. trachomatis* infectious progeny [229]. Although the delivery of CT847 into host cells by *C. trachomatis* has never been shown, this suggests that the CT847:GCIP interaction leads to the destruction of GCIP and that this is beneficial for chlamydial infection of host cells.

### The intriguing CPAF

In addition to Cdu1 and Cdu2 (see above), *C. trachomatis* encodes other proteases and, among them, CPAF (chlamydial protease/proteasome-like activity factor) [231] is probably the most intensively studied chlamydial effector protein [232–237]. Following the previous detection of a chlamydial protease activity within host cell lysates of infected cells [238], CPAF was identified by mass spectrometry after biochemical fractionation of this activity from the cytosol of *C. trachomatis-*infected cells [231]. Subsequently, numerous host cell and chlamydial substrates of CPAF have been identified (reviewed in [239]). However, it was eventually shown that cleavage of most of these proteins by CPAF occurs artificially during the preparation of cell lysates and not within intact infected cells [240]. Nonetheless, biochemical and structural biology studies revealed unambiguously that CPAF is a serine protease produced in the chlamydiae as a 70 kDa protein zymogen [241–244]. *In vitro*, transient concentration-dependent homodimerization of CPAF leads to autocleavage and formation of a stable homodimer [241, 244]. This homodimer undergoes subsequent autocatalytic processing that removes internal inhibitory sequences and leads to the formation of mature activated CPAF [231, 241, 242].

*C. trachomatis* CPAF has been detected in the cytosol of infected host cells by IF microscopy (e.g. [58, 231, 245, 246]). However, depending on the conditions used in the preparation of the samples, CPAF can be detected solely within the lumen of the inclusion by IF microscopy [247]. Regardless of this, CPAF contains a signal peptide recognized by the Sec system that enables protein transport across the bacterial inner membrane [24]. Moreover, in a *C. trachomatis* strain defective for an essential component of the chlamydial T2S system [14], CPAF is retained in an inactive form within the chlamydiae [245]. This indicates that the CPAF activation process likely occurs in the inclusion lumen, after the T2S-dependent transport of CPAF from the chlamydial periplasm. The CPAF activation is regulated by human serine peptidase inhibitor 15 (PI15), which has been shown to localize within the inclusion and to bind CPAF [247]. As mentioned above, there is data indicating that CPAF could be transported into the host cell cytosol. However, the underlying hypothetical secretion mechanism is unknown. Another possibility is that, during *C. trachomatis* host cell infection, active CPAF remains within the inclusion where it might exert its functions until when its cytoplasmic and extracellular targets become accessible by the sequential loss of integrity of the inclusion and host cell plasma membrane.

A *C. trachomatis cpaf* null-mutant reveals a ~2 to 3-fold defect in the generation of infectious progeny [245, 247, 248], indicating a function of CPAF during the chlamydial developmental cycle. Accordingly, possible substrates and functions associated with an activity of CPAF in the inclusion lumen and in host cell cytoplasm have been revealed by characterization of *cpaf* null mutants and/or by using conditions maintaining CPAF inactive in protein extracts from infected cells. A mass spectrometry analysis of lysates from cells infected by a *C. trachomatis cpaf* null mutant and the wild-type CPAF-producing strain, revealed nine chlamydial and six host cell proteins with high likelihood of being less abundant in extracts of cells infected by the CPAF-producing chlamydiae [248]. Remarkably, the chlamydial proteins identified were the five DUF582-containing *C. trachomatis* effectors (see above), the CT847 effector (see above), and three other T3S system-related proteins [248]. CT620 and CT711 are proteolytically processed in cells infected by CPAF-producing *C. trachomatis* [212, 248], and CT620 and CT621 have been detected in the inclusion lumen [212]. It is therefore possible that CT620, CT621, and CT711 are CPAF targets. The 6 host proteins identified are all involved in innate immunity [248]. The CPAF-dependent alterations in the levels of these proteins are related with reduced translocation to the nucleus of cells infected by wild-type *C. trachomatis* of the p65 subunit of NF-κB [248]. This mechanism of inhibition of the host innate immune response by *C. trachomatis* is likely a consequence of the activity of CPAF on other effectors or T3S system-related proteins [248].

Other possible targets of CPAF in the inclusion lumen are *C. trachomatis* OmcB [249], which could be related to the redifferentiation of RBs into EBs [249], and PI15 [247], in the context of regulation of CPAF activity within the inclusion [247]. In the host cell cytoplasm, CPAF cleaves vimentin and lamin-associated protein-1 (LAP1; also known as torsin 1A-interacting protein 1 (TOR1AIP1)), but possibly only upon loss of the integrity of the inclusion membrane [245]. Based on phenotypes displayed in cultured cells infected by *cpaf* null mutant strains, CPAF has been also associated with inhibition of host cell cytokinesis [250] and with *C. trachomatis* lytic exit [251]. Although the activity of CPAF on vimentin and LAP1 could help to promote the lytic exit [245], these CPAF-dependent effects are probably a consequence of its action on other chlamydial proteins [248, 250, 251].

During chlamydial lytic exit, the cytoplasmic and inclusion contents, including CPAF, are also released. Different data support that extracellular CPAF promotes evasion of the host innate immune response [252–254], which could be related with the role of CPAF in helping *C. trachomatis* survival in the mouse lower genital tract [255]. CPAF cleaves the formyl peptide receptor 2 (FPR2) on the surface of neutrophils, and thereby prevents downstream intracellular signalling normally leading to the activation of neutrophils and of their antimicrobial mechanisms [252]. Accordingly, while a *C. trachomatis cpaf* mutant shows defects in mice infection models, these defects are suppressed in neutrophil-depleted or in FPR2-knock-out mice [252]. Furthermore, CPAF can cleave antimicrobial peptides with antichlamydial activity, such as cathelicidin LL-37 [253], and complement factors C3 and B [254].

In summary, *C. trachomatis* secretes a potent serine protease (CPAF) with functions within host cells, during the chlamydial developmental cycle, as well as in the extracellular space, after the release of the chlamydiae from infected cells. CPAF is conserved in *Chlamydiaceae*, and in *Chlamydia*-like organisms infecting amoeba there are homologues showing ~30% of amino acid sequence identity to *C. trachomatis* CPAF [256]. Therefore, some of the functions of CPAF could be conserved in *Chlamydiales*.

### Chlamydial HtrA and Tsp: proteases that also modulate host cell processes?

Chlamydial high temperature requirement protein A (HtrA) [257] and tail-specific protease (Tsp) [258] are other *C. trachomatis* proteases that have been implicated in the subversion of host cell processes.

HtrA serine proteases are conserved in unicellular and multicellular organisms where they mostly play an important role in protein quality control [259]. In Gram-negative bacteria, HtrA proteins are mainly known for functioning in the periplasm, but there is evidence indicating that they can also be transported to the extracellular environment by pathogenic bacteria and have a direct role in host cell subversion [260]. In the case of *C. trachomatis* HtrA, in addition to its expected functions in chlamydial physiology and development [261], the protein has been detected in the host cell cytosol and in the inclusion lumen by IF microscopy [262]. *C. trachomatis* HtrA possesses a signal peptide recognized by the Sec system [262]. Given that HtrA is also detected in the inclusion lumen, the protein could be a T2S substrate. However, besides the detection of *C. trachomatis* HtrA in the host cell cytosol, there is no additional evidence for a possible function of the protein in subverting host cell functions.

Tsps are present in many bacteria where they have diverse housekeeping roles and act by processing the C-terminal region of different target proteins [263]. In *C. trachomatis*, Tsp has a chaperone activity and functions in protein quality control [264]. Although *C. trachomatis* Tsp has never been detected in the host cell cytosol, ectopically expressed Tsp can cleave the p65 subunit of NF-κB [258], and Tsp can bind the human steroid receptor RNA activator 1 (SRA1) protein by yeast two-hybrid and *in vitro* pull-down assays [265]. However, cleavage of p65 is not observed in *C. trachomatis*-infected cells under controlled conditions of cell lysis [240], and the physiological significance of the Tsp:SRAP1 interaction is unclear [264].

### A virulence plasmid-encoded secreted protein

Most *Chlamydia* species possess a cryptic plasmid encoding the so-called plasmid glycoproteins (pGPs 1-8) and two small RNAs [266]. *C. trachomatis* pGP3 has been found associated with the outer membrane of EBs [267], and also in the host cell cytosol and partly in the inclusion lumen [268] **(Figure 4B)**. pGP3 is also found in the cytosol of cells infected by other *Chlamydia* species carrying the virulence plasmid [268]. However, the secretion pathway by which chlamydiae deliver pGP3 into the host cell cytosol is unknown [266, 268].

pGP3 is an immunodominant antigen, but only the native trimeric form of the protein can be recognized by human antibodies [267, 269, 270]. In fact, pGP3 is present as a trimer both in the chlamydial outer membrane and in the host cell cytosol [270]. The 3D structure of the pGP3 trimer has been determined by X-ray crystallography and one of its domains revealed a fold similar to cytokines of the tumor necrosis factor family [271].

The generation and characterization of *C. trachomatis* and *C. muridarum* strains carrying plasmids with the *pgp3* gene inactivated, revealed an important virulence role of pGP3 in mice infection models [272–274]. The attenuation in virulence observed because of the lack of pGP3 was identical to the one observed with plasmidless strains [272, 273]. Experiments with purified pGP3 provided possible explanations for its virulence functions. Purified pGP3 binds to, and neutralizes, cathelicidin LL-37 [275], also a target of CPAF (see above). LL-37 also modulates the immune response, and the binding of pGP3 to this antimicrobial peptide inhibits the LL-37-dependent chemotaxis of neutrophils and cytokine release by epithelial cells [276]. Conversely, pGP3 alone can induce cytokine release by macrophages [268] and neutrophils [276] and, at least in neutrophils, this pro-inflammatory activity is enhanced by the pGP3:LL-37 complex [276]. Purified pGP3 can also inhibit apoptosis in HeLa cells [277]. These pGP3-dependent effects observed with the purified protein could have physiological significance if pGP3 in the host cell cytosol, or in the inclusion lumen, is released into the extracellular milieu after chlamydial lytic exit. Alternatively, pGP3 could potentially mediate the observed effects from the outer membrane of extracellular EBs.

### Additional *C. trachomatis* proteins detected in the host cell cytosol

The *C. trachomatis* proteins CT311 [32] and CT795 [278] were also found in the cytosol of host cells by IF microscopy (**Table 2** and **Figure 4B**). Transport of CT311 and CT795 from the chlamydiae into the host cell cytosol can be prevented by a small molecule inhibitor of the Sec system [32, 278]. As both proteins can also be found in the lumen of the inclusion [32, 278], this suggests that they could be transported from the periplasm by the chlamydial T2S system. How these two proteins can then access the host cell cytosol is unclear. Little is known about possible effector functions of CT311 and CT795. However, CT311 has been shown to also localize in the nucleus of mammalian cells **(Figure 4B)**, either in *C. trachomatis*-infected cells or after its ectopic expression [279].

### Incs with no bilobed hydrophobic region

Among the *C. trachomatis* proteins that have been detected in the inclusion membrane but do not possess a bilobed hydrophobic region, Cap1 and CopN are normally classified as Incs [50, 59].

Cap1 was identified as a protein accessing the host cell cytosol based on a screen for chlamydial proteins recognized by a *C. trachomatis*-specific CD8^+^ T-cell line [280]. Cap1 was then localized to the inclusion membrane [280], which was confirmed in subsequent studies [50, 63]. Little is known about the function of Cap1, but the protein has been associated with host cell LDs (see above) [221].

Several bacteria harbouring T3S systems have a so-called gatekeeper protein required for secretion of the T3S system translocators and controlling the beginning of effector delivery into host cells, which can involve secretion of the gatekeeper itself [281]. CopN is the likely *C. trachomatis* T3S system gatekeeper, and it was the first chlamydial protein shown to be a T3S substrate [23]. It was localized in the inclusion membrane [23], which was confirmed in a subsequent study [50]. This localization of *C. trachomatis* CopN could be strictly associated with its regulatory function, but a possible effector role should not be disregarded. For example, *C. pneumoniae* CopN has been shown to interfere with host cell microtubules [282, 283]; however, *C. trachomatis* CopN does not display this activity on microtubules [282].

## *C. TRACHOMATIS* PROTEINS SECRETED INTO THE LUMEN OF THE INCLUSION

In addition to the *C. trachomatis* proteins that localize in the host cell cytoplasm or nucleus, or in the inclusion membrane, and that are also found in the lumen of the inclusion (GlgX, CT311, CT620, CT621, CT622, CT795, GlgA, HtrA, CPAF, and pGP3; **Table 2)**, there are at least six *C. trachomatis* proteins that localize within the lumen of the inclusion and which thus far have not been localized in the host cell cytoplasm (Pls1, Pls2, CT142, CT143, CT144, and Ptr; **Table 2)**. Furthermore, in addition to GlgX and GlgA, there is indirect functional evidence for a localization of other *C. trachomatis* glycogen metabolizing enzymes in the inclusion lumen.

### The glycogen metabolizing enzymes

**Figure 5:**
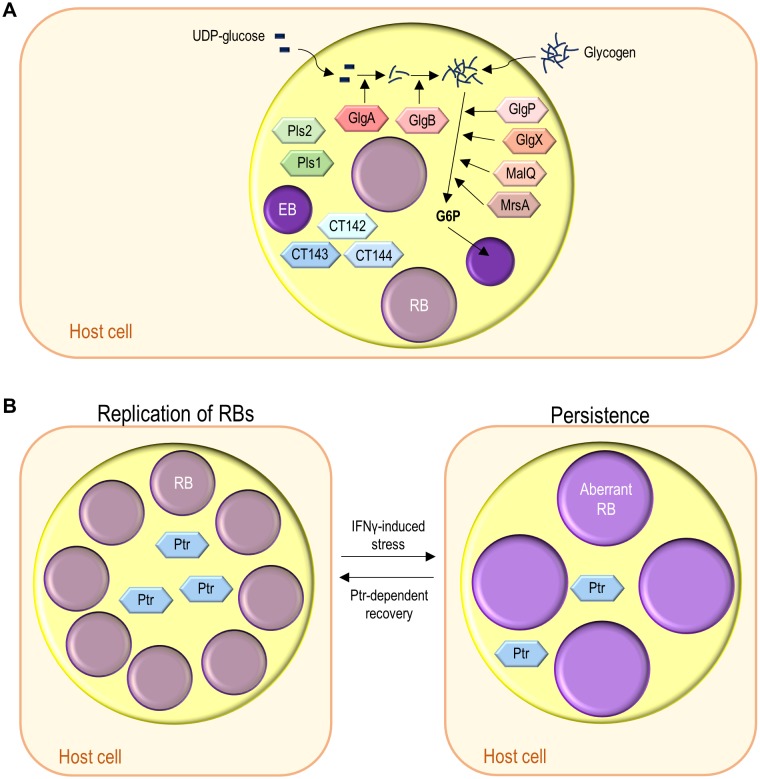
FIGURE 5: *C. trachomatis* proteins secreted into the inclusion lumen. **(A)** *C. trachomatis* glycogen metabolizing enzymes shown (GlgA and GlgX) or deduced (GlgB, GlgP, MalQ, and MrsA) to localize in the inclusion lumen, and *C. trachomatis* proteins (Pls1, Pls2, CT142, CT143, and CT144) of unknown function appearing in the inclusion lumen, by IF microscopy, as large globular structures; G6P, glucose 6-phosphate. **(B)** *C. trachomatis* putative protease Ptr that specifically mediates the recovery from IFNγ-induced chlamydial persistence. See list of abbreviations, main text, and **Table 2** for details.

*C. trachomatis* possesses the genes encoding the proteins required for glycogen synthesis (GlgA, GlgB, GlcC) and hydrolysis (GlgX, GlgP, and MalQ) [21]. For glycogenesis, GlgC converts glucose 1-phosphate into ADP-glucose, which is then used by the glycogen synthase GlgA to generate a linear chain of α1,4-linked glucose molecules; lastly, the branching enzyme GlgB produces glycogen from α1,4-linked glucose. For glycogenolysis, the activity of GlgX, GlgP and MalQ degrades glycogen into glucose 1-phosphate **(Figure 5A)**.

There is a *Chlamydia* virulence plasmid-dependent accumulation of glycogen in the lumen of the inclusion of *C. trachomatis* and *C. muridarum* [284, 285]. The plasmid-dependency is partially explained because plasmid-encoded pGP4 is required for the normal expression of *glgA* [286, 287]. Furthermore, GlgA can be detected in the inclusion lumen and in the host cell cytosol by IF microscopy [288], and there is evidence that it can be imported from the host cell cytosol into the inclusion lumen [289]. In addition, GlgA and GlgB, but not GlgC, possess a T3S signal recognized by *S. flexneri* [289], and *C. trachomatis* strains with mutations in GlgB show an accumulation of aggregates within the inclusion possibly corresponding to unbranched glycogen [14]. Altogether, this indicates that GlgA and GlgB should function in the inclusion lumen **(Figure 5A)**. Differently from other bacterial glycogen synthases, chlamydial GlgA can use both ADP- and UDP-glucose as substrate [289]. Accordingly, in *C. trachomatis*-infected cells, the host transporter SLC35D2 is recruited to the inclusion membrane and likely mediates the transport of UDP-glucose into the lumen of inclusion [289] **(Figure 5A)**. Thus, *C. trachomatis* should mediate the T3S-dependent transport of GlgA and GlgB into the inclusion lumen, and the ability of GlgA to use UDP-glucose as substrate enables the chlamydiae to synthesize glycogen in this confined compartment **(Figure 5A)**. However, the *C. trachomatis* T2S system should also play a role in the chlamydial glycogen metabolism because a strain with a mutation in an essential component of this protein transport system shows abnormal glycogen aggregates within the inclusion [14]. Finally, the accumulation of glycogen in the *C. trachomatis* inclusion also depends, but to a lesser extent, on its transport in bulk from the host cell cytosol **(Figure 5A)**, which brings along the host glycogen synthase Gys1 [289].

It is unclear how the accumulation of glycogen within the inclusion benefits chlamydiae. By one side, a plasmidless *C. trachomatis* strain and a *glgB* mutant do not show a defect in the generation of infectious progeny [14, 286]. On the other side, depletion of the host glycogen synthase Gys1 leads to a reduction in the production of *C. trachomatis* and *C. muridarum* infectious progeny, and *C. muridarum* strains with mutations in GlgA are defective in the generation of infectious progeny [289]. *C. trachomatis* GlgP, GlgX, and MalQ also possess a T3S signal recognized by *S. flexneri* and at least GlgX has been detected in the inclusion lumen and membrane by IF microscopy [289]. Therefore, *C. trachomatis* should be able to hydrolyse glycogen in the inclusion lumen into glucose 1-phosphate **(Figure 5A)**. Because EBs do not uptake glucose 1-phosphate [289], its conversion into glucose 6-phosphate (which EBs can uptake) might be performed within the inclusion by a *C. trachomatis* phosphoglucomutase (MrsA), which also possesses a T3S signal recognized by *S. flexneri* [289] **(Figure 5A)**.

### A secreted protein mediating recovery from stress-induced persistence

In cultured cells infected by *C. trachomatis*, nutrient deprivation, or exposure to certain cytokines or antibiotics, can lead to the reversible formation of non-dividing aberrant RBs, a persister-like chlamydial form (reviewed in [290]) **(Figure 1)**, which might be important *in vivo* [290, 291]. A genetic screen for *C. trachomatis* mutants showing defects in recovery from stress-induced persistence, identified the gene encoding a putative protease, Ptr [292]. A *C. trachomatis ptr* null mutant shows a defect in recovery from IFNγ-induced stress, but not from penicillin-induced stress [292] **(Figure 5B)**. By IF microscopy, Ptr was found in the inclusion lumen outside of the chlamydiae [292] **(Figure 5B)**. Ptr possesses a putative signal peptide recognized by the Sec system [292], and its localization in the inclusion lumen suggests it should be a T2S substrate. However, the mechanism by which Ptr functions is currently unknown.

### Proteins of unknown function secreted into the inclusion lumen

*C. trachomatis* encodes proteins showing similarity to the autotransporter passenger domain of chlamydial PmpC that were named Pmp-like secreted (Pls) proteins [293]. By IF microscopy, Pls1 and Pls2 were localized to the inclusion lumen, where they appear as globular structures outside of the chlamydiae [293] **(Figure 5A)**. Pls1 and Pls2 lack obvious Sec-dependent signal peptides [293]. Thus, it is currently unclear how they are secreted into the lumen of the inclusion [293]. Microinjection of *C. trachomatis*-infected cells with anti-Pls1 and anti-Pls2 antibodies inhibits inclusion expansion, suggesting that Pls1 and Pls2 could be important for *C. trachomatis* and/or inclusion growth [293].

The *C. trachomatis* genes *ct142, ct143*, and *ct144* genes are organized in an operon encoding three T3S substrates [227, 294]. Strikingly, by IF microscopy, CT142, CT143, and CT144 appear in globular structures outside of chlamydiae resembling those seen with anti-Pls1 and anti-Pls2 antibodies [293–295] **(Figure 5A)**. Furthermore, CT142, CT143, and CT144 co-localize with each other within the inclusion lumen, suggesting that they could be part of protein complexes [294]. Purified CT143 can induce pro-inflammatory cytokine secretion by THP1 macrophages [296], but the significance of this finding is unclear.

In summary, Pls1, Pls2, CT142, CT143, and CT144 have unknown function but they are secreted into the lumen of the inclusion where they appear as globular structures, as detected by IF microscopy **(Figure 5A)**. Coincidently, the genes encoding Pls1, Pls2, CT142, CT143, CT144, and also the glycogen synthase GlgA (see above), are amongst the *C. trachomatis* chromosomal genes more clearly upregulated by the *Chlamydia* virulence plasmid through pGP4 [287, 295].

## CONCLUSIONS AND OUTLOOKS

*C. trachomatis* has been shown to deliver at least ~60 proteins into the inclusion membrane and cytoplasm of host cells that function as effectors **(Tables 1 and 2)**. Moreover, there are numerous putative Incs that have never been localized to the inclusion membrane [42, 43], and many candidate chlamydial T3S substrates whose characterization has not been further reported [63, 227]. Even if several of these proteins could be false hits, the actual number of *C. trachomatis* proteins delivered into the inclusion membrane and host cell cytoplasm might be ~ 70-90. In addition, *C. trachomatis* also secretes proteins into the inclusion lumen (**Table 2** and **Figure 5**). Considering that a typical *C. trachomatis* genome encodes ~ 900 proteins [21], > 7%, and possibly ~10%, of the coding capacity is devoted to produce effector proteins acting outside of the chlamydiae, in the host cell cytoplasm, within the inclusion lumen, and/or extracellularly.

A feature of pathogenic bacteria delivering high numbers of effectors into host cells is redundancy, which can be, for example, effectors with similar activity and functions or effectors with different activity but targeting the same host cell process [297]. Considering that *C. trachomatis* genomes are relatively small (~ 1 Mb) and underwent extensive reductive evolution [21, 298], it could be expected that redundancy might be less pronounced than what is observed in other intracellular pathogens, such as in *Legionella pneumophila* [297]. However, thus far, no *C. trachomatis* effector gene has been shown to be essential, and the analysis of some effector gene mutants suggests possible redundancy. Overall, this indicates that some type of redundancy might also be relevant among chlamydial effectors.

Another feature of pathogenic bacteria carrying several effector genes is that some of them were acquired through horizontal gene transfer [299–301]. Given the obligate intracellular nature of *Chlamydia*, horizontal gene transfer is less important, but not irrelevant [302], in chlamydial evolution than in other bacterial pathogens. For example, pathogenicity islands are virtually non-existent in the genome of *C. trachomatis*. However, genes encoding Inc proteins and other T3S substrates have been shown to be encoded within operons [51, 186, 294]. Apart from suggesting a related function of effectors encoded within the same operon, the reasons beyond this genetic organization only in some cases is presently unclear.

### Timely transport of *C. trachomatis* proteins into the inclusion membrane and host cell cytoplasm

*C. trachomatis* delivers at least six effector proteins (TarP, TmeA, TmeB, TepP, and possibly CT166 and CT622) into the cytoplasm of host cells from extracellular adhering EBs and/or shortly after their internalization **(Figure 4A)**. Some of these effectors are important for chlamydial invasion (TarP, CT622, and TmeA), host cell survival (TarP) and immune signalling (TepP). Furthermore, TarP [168, 186], CT622 [200, 201], TmeA [186, 189], and TmeB [186] have been detected in the host cell cytoplasm several hours after invasion; therefore, they should have additional functions other than during chlamydial entry. It is possible that, similarly to *Salmonella* effector proteins [303], some of the *C. trachomatis* effectors packed in EBs downmodulate the alterations in the actin cytoskeleton that promote chlamydial invasion. An obvious candidate is CT166, which can potentially inactivate the TarP-dependent activation of RAC1. However, while the activity of *Salmonella* effectors causes reversible changes in Rho family proteins [303], the glucosylating activity of CT166 on RAC1 should be irreversible. Furthermore, CT166 is not expressed by all *C. trachomatis* strains. Another key aspect that remains unclear is how the nascent inclusion limits interactions with the endosomal pathway to prevent chlamydial destruction in a phagolysosome. This early subversion of the endocytic pathway occurs even in the absence of chlamydial protein synthesis [304]. Therefore, the relevant effector(s) are surely among the proteins packed in EBs [159].

After *C. trachomatis* uptake by host cells, chlamydial gene expression leads to the production and secretion of effector proteins that influence the subsequent stages of the developmental cycle. This group of effectors is largely comprised by early-cycle Incs, which modulate interactions with host cell vesicular trafficking (IncE and CpoS), help the migration of the nascent inclusion to the centrosomal region (CT850), establish ER-inclusion MCSs and promote the non-vesicular transport of sphingomyelin into the inclusion (IncV and IncD), and mediate inclusion membrane stability (CpoS, IncC, CT383). IncV might even be delivered earlier into the inclusion membrane as it is present in EBs [159]. A detailed understanding of how *C. trachomatis* modulates these processes remains to be established. For example, it is still unclear how the inclusion intercepts vesicular trafficking from the TGN to selectively acquire sphingolipids and cholesterol [115–117, 305], and exactly how it continues to avoid fusion with hydrolytic-rich lysosomes [304, 306].

As the developmental cycle continues, several other chlamydial effectors are delivered into the cytoplasm of host cells. These effectors mediate fusion between inclusions (IncA), continue subverting host cell vesicular trafficking (IncA, and perhaps CteG and DUF582-containing CT619, CT620, CT621, CT711, and CT712), modulate microtubules (IPAM) and mediate their modification (InaC), promote the assembly of F-actin (InaC) and the redistribution of the Golgi around the inclusion (InaC, Cdu1 and Cdu2), and possibly promote the acquisition of LDs by the inclusion (Lda1, Lda2, and Lda3), interfere with host cell transcription (NUE), and modulate host cell death (CpoS and Cdu1). Finally, chlamydial host cell exit is also controlled by Incs (MrcA and CT228), and by the *Chlamydia* virulence plasmid, likely through the regulation of expression of T3S effectors and/or of CPAF [251, 295]. Even after chlamydial exit from infected cells, some *C. trachomatis* effectors concomitantly released from the inclusion lumen and/or host cell cytosol continue to function extracellularly, as is the case of evasion of the innate immune response by CPAF, and possibly by pGP3. Evidently, much remains to be understood about the subversion of all these processes by *C. trachomatis*.

### The inclusion lumen: more than just housing chlamydiae

The inclusion lumen is a functionally and metabolically important compartment of *C. trachomatis*-infected host cells. For this, *C. trachomatis* secretes proteins into the inclusion lumen synthesizing and hydrolysing glycogen [289], mediating chlamydial recovery from IFNγ-induced stress [292], with unknown function, and/or perhaps *en route* to the inclusion membrane or host cell cytoplasm. Furthermore, even if chemical fixation in preparation of samples for microscopy can create artifacts [307], host cell cytoplasmic glycogen [289], different host cell proteins [223, 247, 289, 308, 309], and LDs [220] and peroxisomes [310] have been detected within the lumen of the inclusion. The mechanisms involved in the transport of these large host cell molecules and organelles into the inclusion are largely unknown. Some of the chlamydial proteins found in the inclusion lumen have also been detected in the host cell cytoplasm (CT311, CT620, CT621, CT622, CT795, GlgA, HtrA, CPAF, and pGP3). It is unclear whether this is due to partial leakage from the T3S system, to intermediates in the transport to the host cell cytoplasm, or if these proteins have functions in both the inclusion lumen and host cell cytosol. It has been suggested that outer membrane vesicles could be involved in the transport of CT311, CT795, HtrA and CPAF from the periplasm, passing through the inclusion lumen into the host cell cytosol [24, 32, 239, 262, 278]. However, as shown for CPAF [14] and deduced by analogy for CT311 and CT795, they are possibly transported into the inclusion lumen by the *C. trachomatis* T2S system. Assuming this is the case, it is unknown how these proteins can reach the host cell cytoplasm before the permeabilization of the inclusion membrane that precedes chlamydial lytic exit.

### The importance and some particularities of the *C. trachomatis* T3S system

Most, if not all, of the proteins delivered by *C. trachomatis* into the inclusion membrane and host cell cytoplasm are transported by the T3S system. *C. trachomatis* EBs appear to be better equipped for T3S than RBs [159], suggesting that this protein transport pathway might be mostly operative before the completion of the EB to RB transition and after the RB to EB re-differentiation. In the T3S pathway, effector proteins travel through a conduit formed by an export apparatus in the bacterial inner membrane linked to a hollow needle-like structure extending from the bacterial surface and connected to a pore complex formed by type III secreted translocator proteins in a target cell membrane [20] **(Figure 2)**. Surprisingly, some *C. trachomatis* T3S substrates are transported into the inclusion lumen [289, 294]. Considering the T3S pathway, it is presently unknown how such chlamydial T3S-dependent transport into the inclusion lumen can occur. Along the same line, an outstanding question is how Incs are inserted into the inclusion membrane. At least two models can be conceived: i) Incs are first transported into the host cell cytosol and then inserted into the inclusion membrane; ii) Incs are delivered directly into the inclusion membrane by lateral partition through the T3S system translocon pore. There is currently no data to support one model or the other. There is also the general question of how T3S effectors containing transmembrane segments, such as Incs, are not targeted to the bacterial inner membrane [311]. Finally, it is likely that a *C. trachomatis* mutant with an essential T3S system gene inactivated would be non-viable. Therefore, although a formality in most cases, final demonstration that Incs and other chlamydial proteins are T3S substrates will require the generation of a chlamydial conditional mutant, which has not been described yet.

### Final remarks

*C. trachomatis* delivers many effector proteins into host cells, and into the lumen of the inclusion, and these proteins interfere with a wide diversity of host cell processes to promote chlamydial invasion, survival, growth, development and dissemination. Although enormous progress has been achieved in recent years elucidating the functions of these chlamydial secreted proteins, we are most likely only seeing the tip of the iceberg. The generation of *C. trachomatis* mutants on relevant genes and their careful characterization in infected cells and in animal models of infection, together with molecular, cell and structural biology studies, will certainly provide explanations to some of the current outstanding issues and originate new concepts as well as additional questions. It might also establish a relation between effector function and *C. trachomatis*-related pathogenicity. This will expand our fundamental understanding of host-pathogen interactions and of chlamydial and host cell biology, which is critical to devise novel prophylactic and therapeutic approaches against infectious diseases.

## Abbreviations:

3D: – three-dimensional,
co-IP: – co-immunoprecipitation,
DUB: – deubiquitinase,
DUF582: – domain of unknown function 582,
EB: – elementary body,
ER: – endoplasmic reticulum,
ESCRT: – endosomal sorting complexes required for transport,
F-actin: – filamentous-actin,
FFAT: – two phenylalanines in an acidic tract,
G-actin: – globular-actin,
GGTases: – geranylgeranyl transferases,
IF: – immunofluorescence,
IFN: – interferon,
Inc: – inclusion membrane protein,
LD: – lipid droplet,
MCS: – Membrane contact site,
MOMP: – major outer membrane protein,
MVB: – multivesicular body,
PIP2: – phosphatidylinositol (4,5)-bisphosphate,
PIP3: – phosphatidylinositol (3,4,5)-trisphosphate,
Pmp: – polymorphic membrane protein,
RB: – reticulate body,
siRNA: – small-interfering RNA,
SLD: – SNARE-like domain,
SNARE: – soluble N-ethylmaleimide-sensitive factor attachment protein receptor,
SH2: – SRC homology 2,
T2S: – type II secretion,
T3S: – type III secretion,
TGN: – trans-Golgi network,
mRNA: – messenger RNA,
VAMP: – vesicle-associated membrane protein.
